# Supplementary material for: Unprecedented Neoverrucosane and Cyathane Diterpenoids with Anti-Neuroinflammatory Activity from Cultures of the Culinary-Medicinal Mushroom Hericium erinaceus
Source: Molecules. 2023 Aug 31;28(17):6380. doi: 10.3390/molecules28176380 (PMC10489798; doi:10.3390/molecules28176380)
Supplement: Supplementary file 1 [file molecules-28-06380-s001.zip › supplementary materials.pdf]

SUPPLEMENTARY MATERIAL

Unprecedented neoverrucosane and cyathane diterpenoids with anti-neuroinflammatory activity from cultures of the culinary-medicinal Mushroom *Hericium erinaceus*

Jing Wei[1 3], Jia-yao Li[2], Xi-long Feng[2], Yilin Zhang[1], Xuansheng Hu[1], Heping Hui[1], Xiaodong Xue[1], Jianzhao Qi[2 *]

[1] College of Biology Pharmacy & Food Engineering, Shangluo University, Shangluo, Shaanxi, China

[2] Shaanxi Key Laboratory of Natural Products & Chemical Biology, College of Chemistry & Pharmacy, Northwest A&F University, 3 Taicheng Road, Yangling 712100, Shaanxi

[3] Qinba Mountains of Bio-Resource Collaborative Innovation Center of Southern Shaanxi Province, Hanzhong, Shaanxi, China

* Corresponding author: qjz@nwafu.edu.cn

Content

Figure S1. HRESIMS spectrum of compound 1.......1
Figure S2. ^{1}H NMR spectra of compound 1 in CD_3OD. 2
Figure S3. ^{13}C spectra of compound 1 in CD_3OD.......2
Figure S4. HSQC spectra of compound 1 in CD_3OD.......3
Figure S5. ^{1}H-^{1}H COSY spectra of compound 1 in CD_3OD. 3
Figure S6. HMBC spectra of compound 1 in CD_3OD.......4
Figure S7. NOESY spectra of compound 1 in CD_3OD.......4
Figure S8. IR spectra of compound 1. 5
Figure S9. UV spectra of compound 1.......5
Figure S10.HRESIMS spectrum of compound 2.......6
Figure S11. ^{1}H NMR spectra of compound 2 in $CDCl_3$. 7
Figure S12. ^{13}C NMR spectra of compound 2 in $CDCl_3$. 7
Figure S13. HSQC spectra of compound 2 in $CDCl_3$.......8
Figure S14. ^{1}H-^{1}H COSY spectra of compound 2 in $CDCl_3$. 8
Figure S15. HMBC spectra of compound 2 in $CDCl_3$.......9
Figure S16. NOESY spectra of compound 2 in $CDCl_3$.......9
Figure S17.IR spectrum of compound 2.......10
Figure S18.UV spectrum of compound 2. 10
Figure S19. ^{1}H NMR spectra of compound 3 in $CDCl_3$ 11
Figure S20. ^{13}C spectra of compound 3 in $CDCl_3$.......11
Figure S21.HRESI-MS spectrum of compound 3. 12
Figure S22. ^{1}H NMR spectra of compound 4 in $CDCl_3$ 13
Figure S23. ^{13}C spectra of compound 4 in $CDCl_3$.......13
Figure S24.HRESI-MS spectrum of compound 4. 14
Figure S25. ^{1}H NMR spectra of compound 5 in CD_3OD 15
Figure S26. ^{13}C spectra of compound 5 in CD_3OD.......15
Figure S27.HRESI-MS spectrum of compound 5. 16
Figure S28. ^{1}H NMR spectra of compound 6 in $CDCl_3$. 17
Figure S29. ^{13}C spectra of compound 6 in $CDCl_3$.......17
Figure S30. ESI-MS spectrum of compound 6. 18
Figure S31. ^{1}H NMR spectra of compound 7 in CD_3OD. 19
Figure S32. ^{13}C spectra of compound 7 in CD_3OD.......19
Figure S33. ESI-MS spectrum of compound 7. 20
Figure S34. ^{1}H NMR spectra of compound 8 in $CDCl_3$. 21
Figure S35. ^{13}C spectra of compound 8 in $CDCl_3$.......21
Figure S36. ESI-MS spectrum of compound 8. 22
Figure S37. ^{1}H NMR spectra of compound 9 in $CDCl_3$. 23
Figure S38. ^{13}C spectra of compound 9 in $CDCl_3$.......23
Figure S39.HRESI-MS spectrum of compound 9. 24
Figure S40. ^{1}H NMR spectra of compound 10 in CD_3OD. 25
Figure S41. ^{13}C spectra of compound 10 in CD_3OD.......25
Figure S42.ESI-MS spectrum of compound 10. 26

Figure S43. ^{1}H **NMR spectra of compound 11 in** CD_3OD**.** 27
Figure S44. ^{13}C **spectra of compound 11 in** CD_3OD**.** 27
Figure S45.ESI-MS spectrum of compound 11. 28
Figure S46. ^{1}H **NMR spectra of compound 12 in** CD_3OD**.** 29
Figure S47. ^{13}C **spectra of compound 12 in** CD_3OD**.** 29
Figure S48.ESI-MS spectrum of compound 12. 30
Figure S49. ^{1}H **NMR spectra of compound 13 in** CD_3OD**.** 31
Figure S50. ^{13}C **spectra of compound 13 in** CD_3OD**.** 31
Figure S51.HRESI-MS spectrum of compound 13. 32
Figure S52.Region of protein instability identified by RMSF analysis. 32
Table S1. IC_{50} **data of 1-9 inhibited the LPS-induced NO production in culture medium.** 33

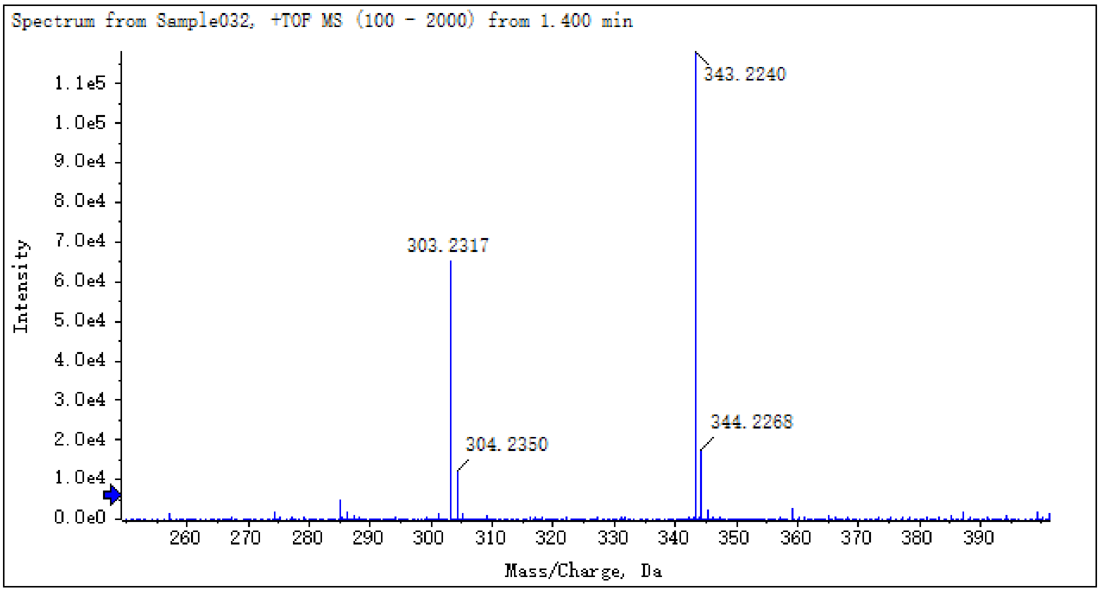

Figure S1. HRESIMS spectrum of compound 1.

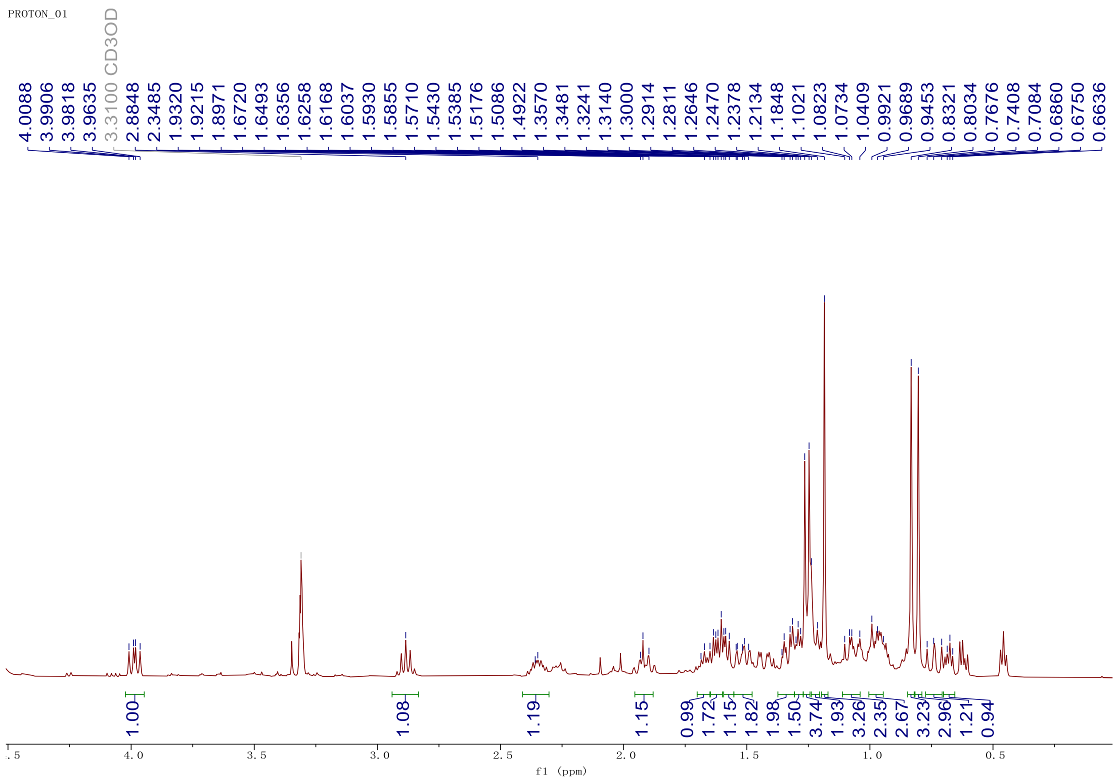

Figure S2. ^{1}H NMR spectra of compound 1 in CD_3OD.

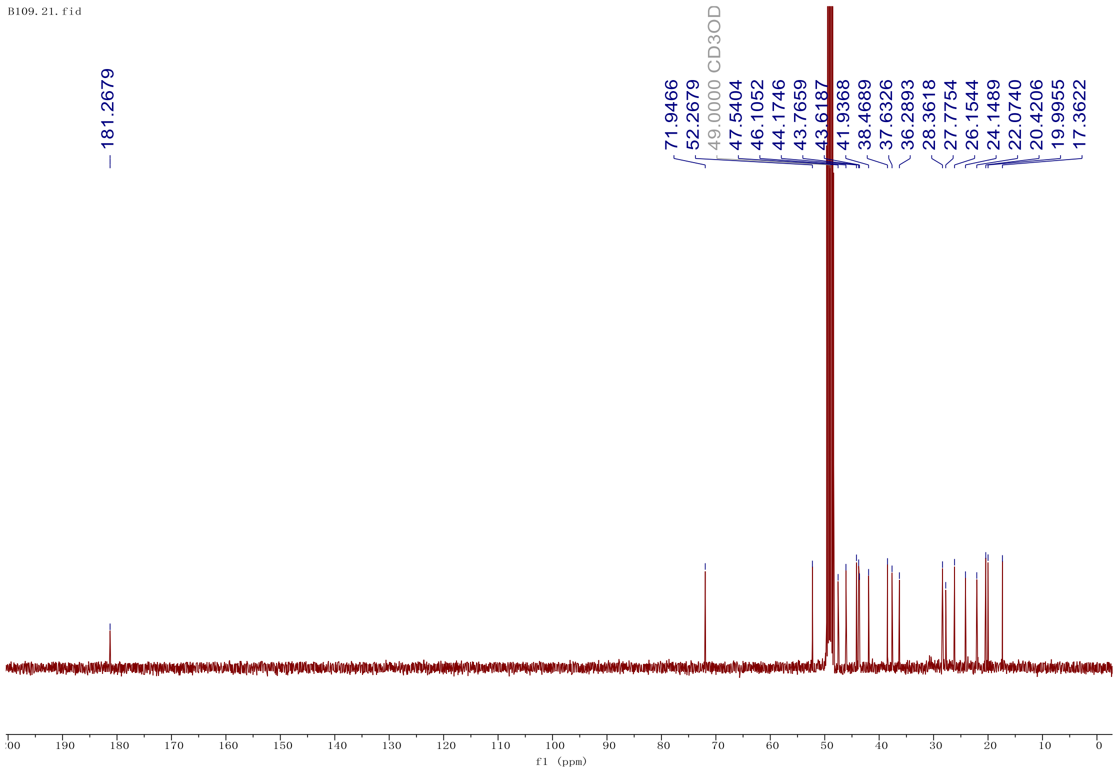

Figure S3. ^{13}C spectra of compound 1 in CD_3OD.

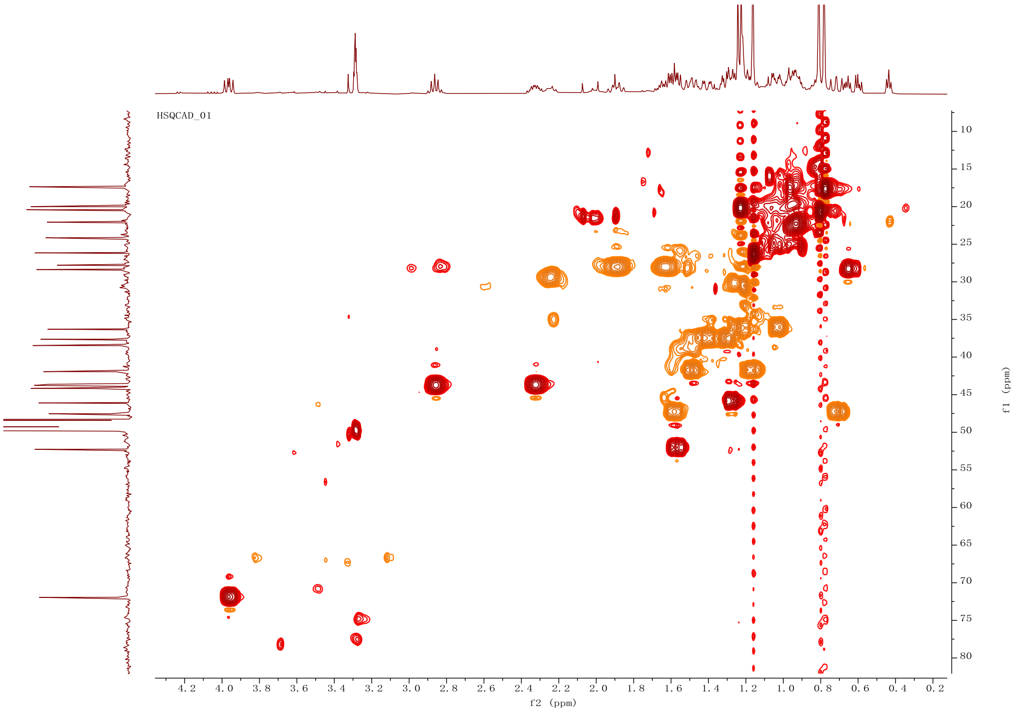

Figure S4. HSQC spectra of compound 1 in CD_3OD.

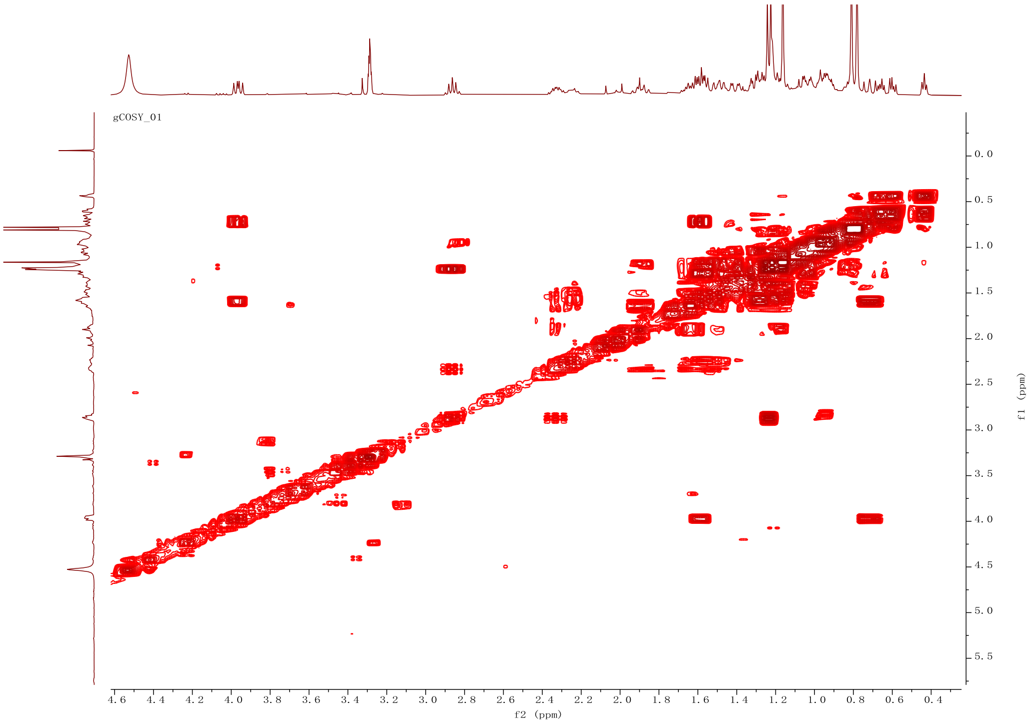

Figure S5. ^1H-^1H COSY spectra of compound 1 in CD_3OD.

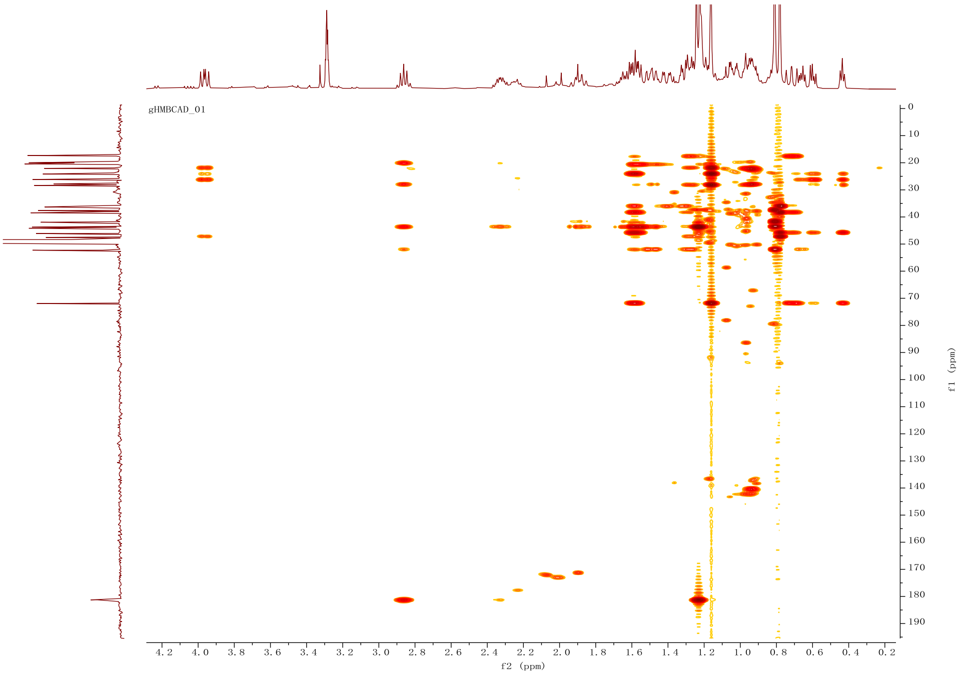

Figure S6. HMBC spectra of compound 1 in CD_3OD.

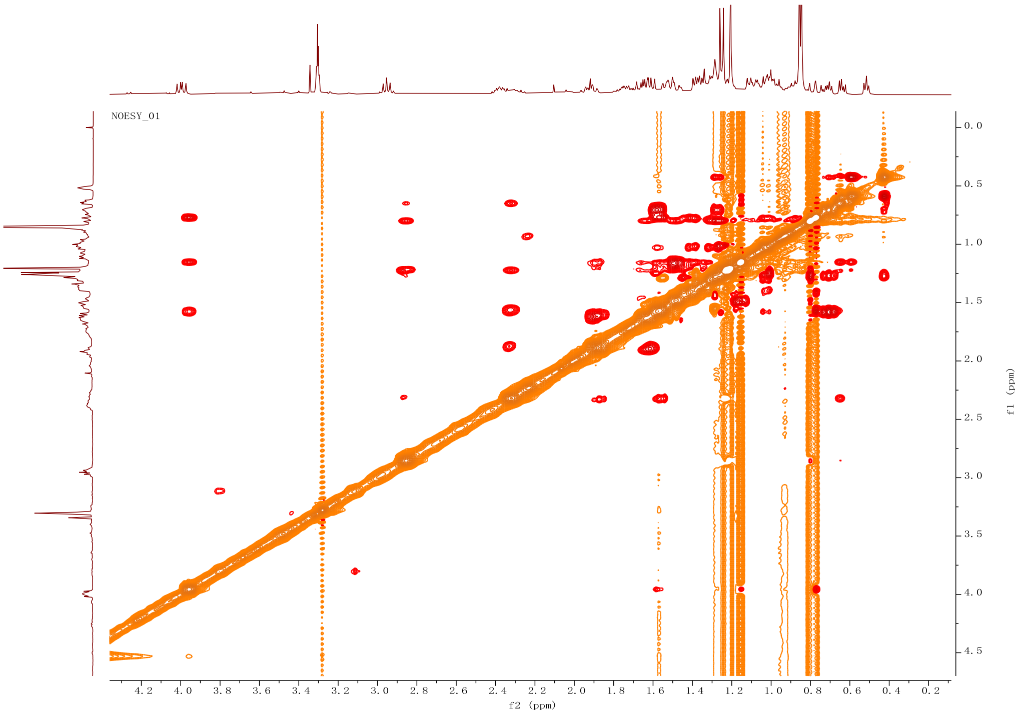

Figure S7. NOESY spectra of compound 1 in CD_3OD.

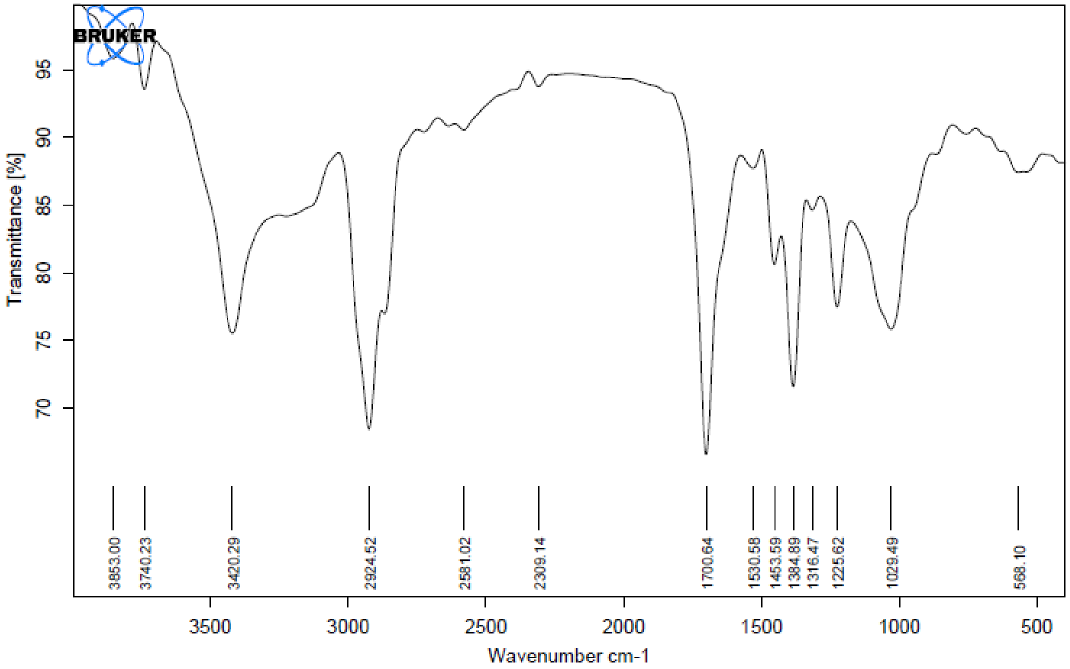

Figure S8. IR spectra of compound 1.

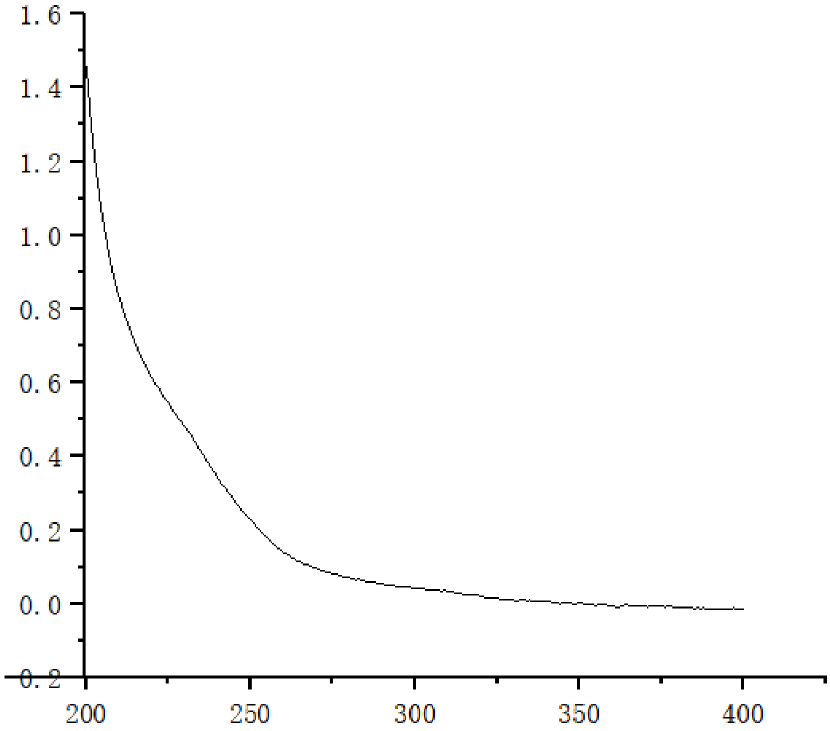

Figure S9. UV spectra of compound 1.

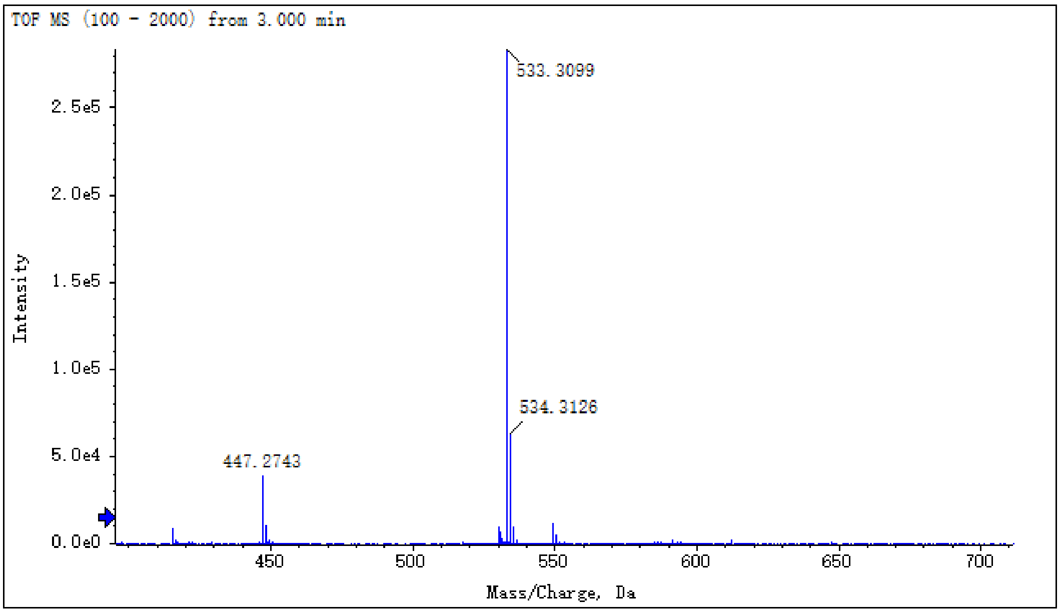

Figure S10.HRESIMS spectrum of compound 2.

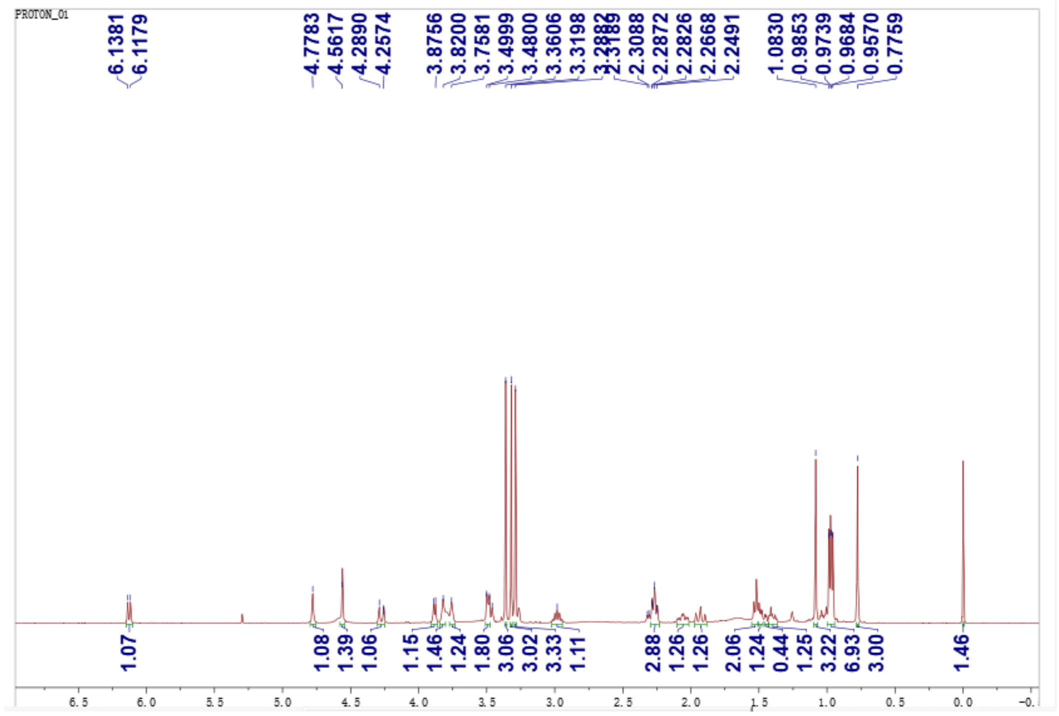

Figure S11. ^{1}H NMR spectra of compound 2 in $CDCl_3$.

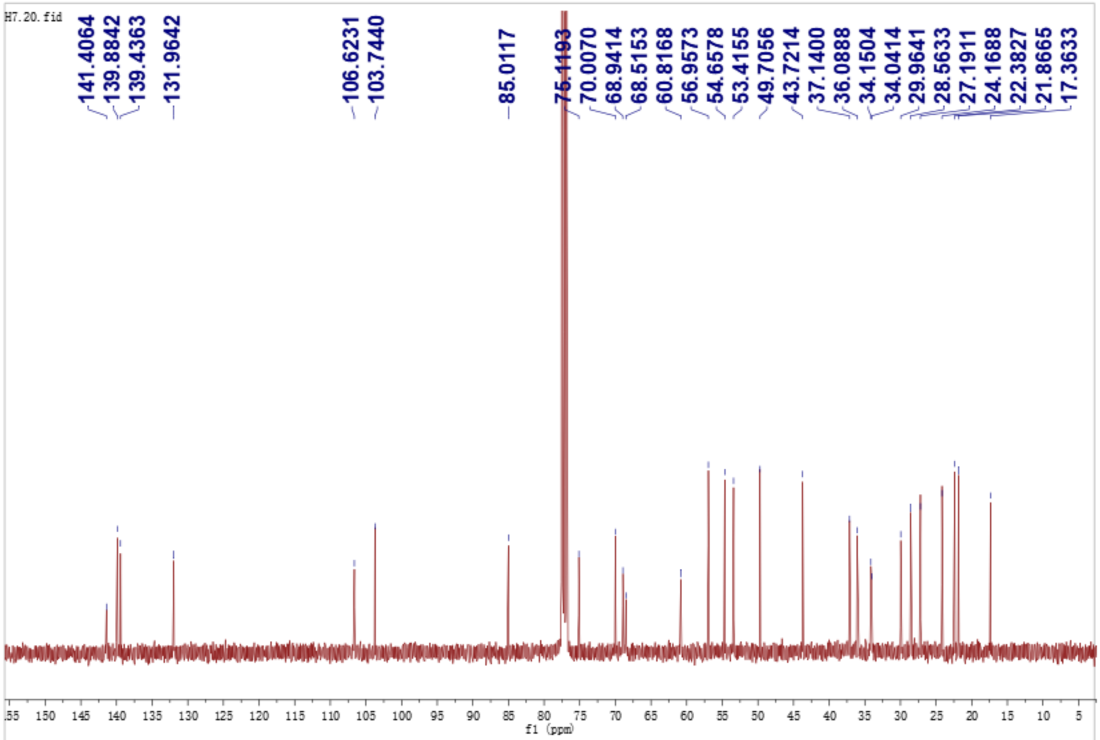

Figure S12. ^{13}C NMR spectra of compound 2 in $CDCl_3$.

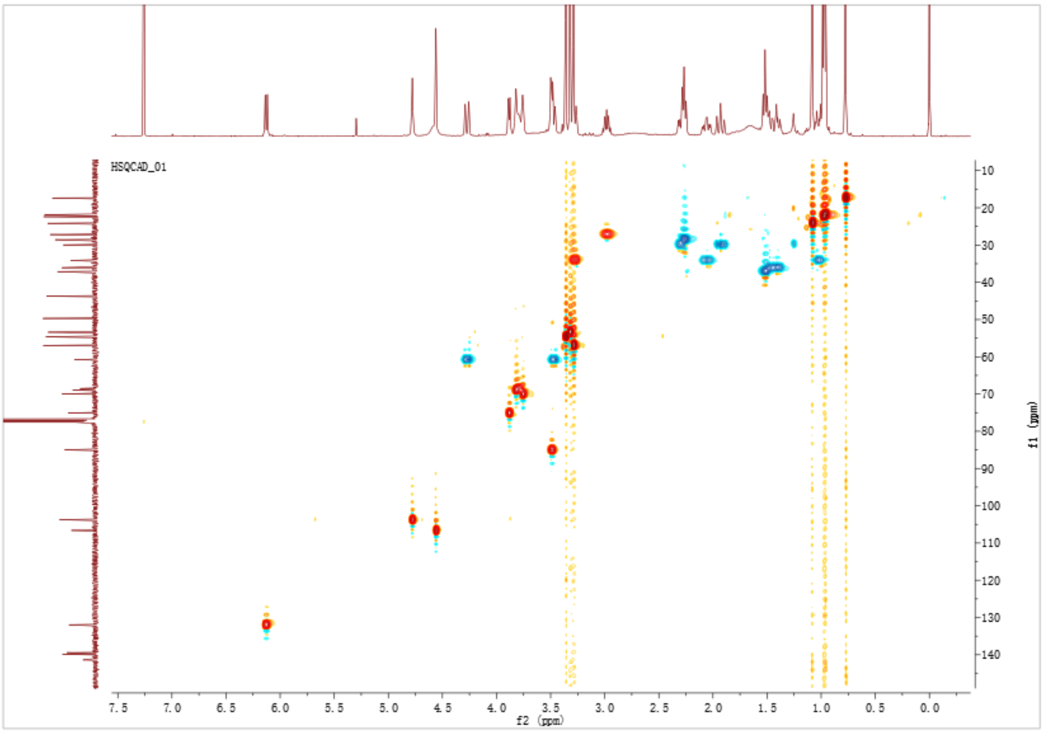

Figure S13. HSQC spectra of compound 2 in $CDCl_3$.

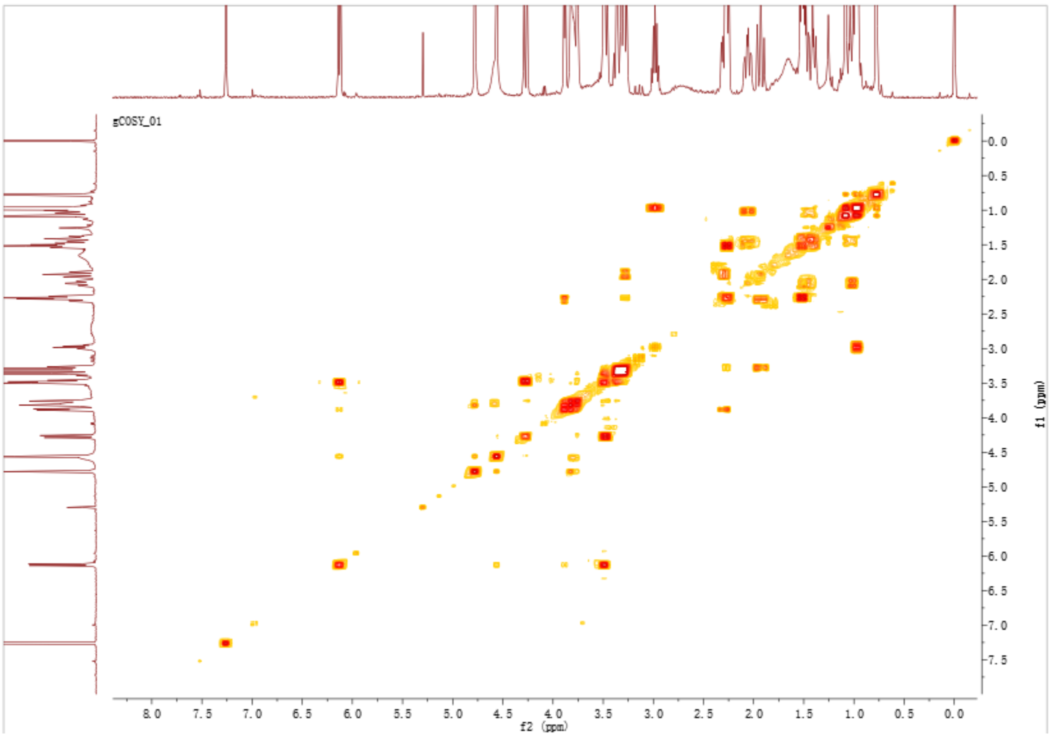

Figure S14. ^1H-^1H COSY spectra of compound 2 in $CDCl_3$.

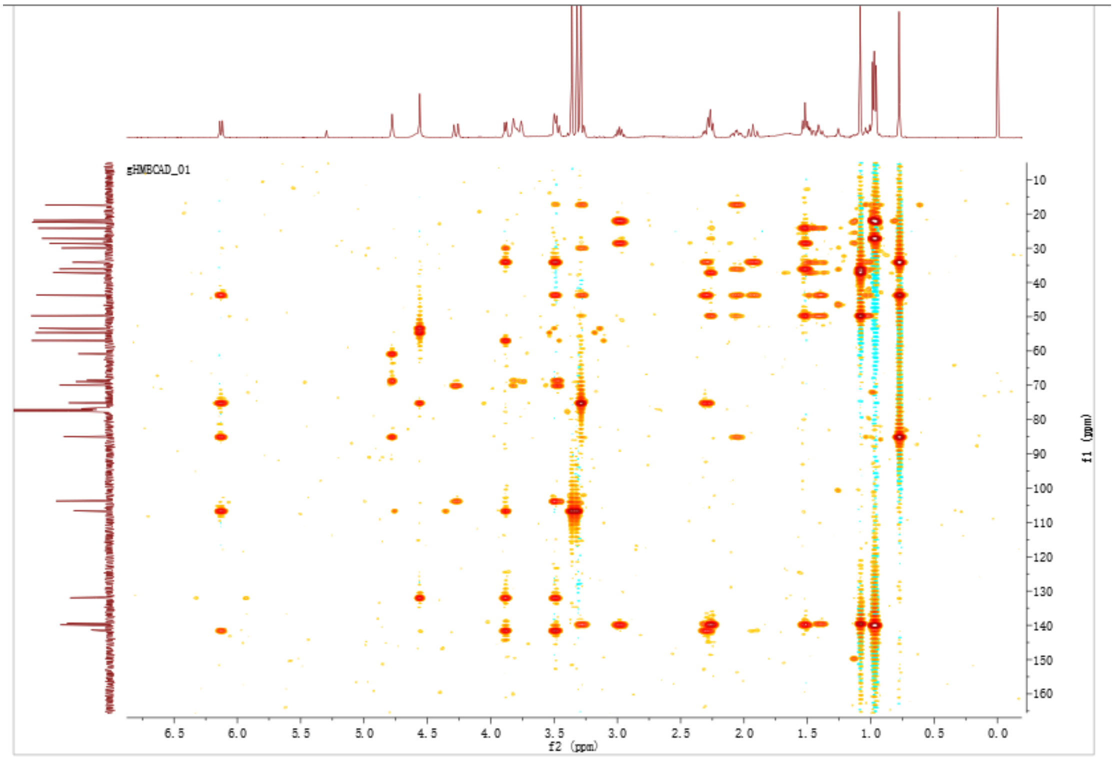

Figure S15. HMBC spectra of compound 2 in $CDCl_3$.

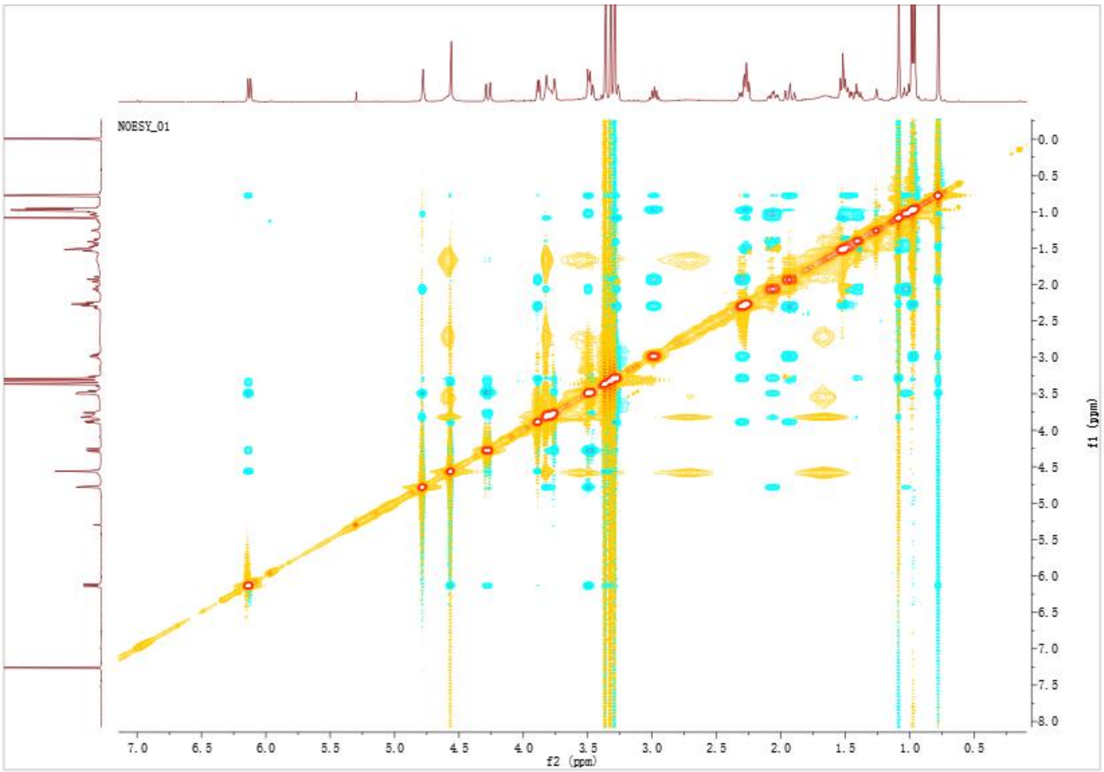

Figure S16. NOESY spectra of compound 2 in $CDCl_3$.

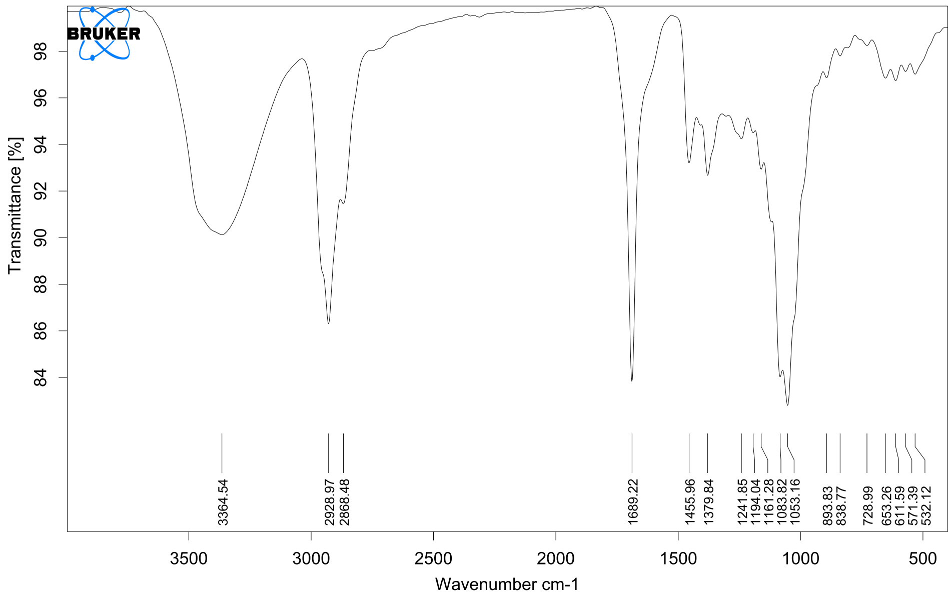

Figure S17.IR spectrum of compound 2.

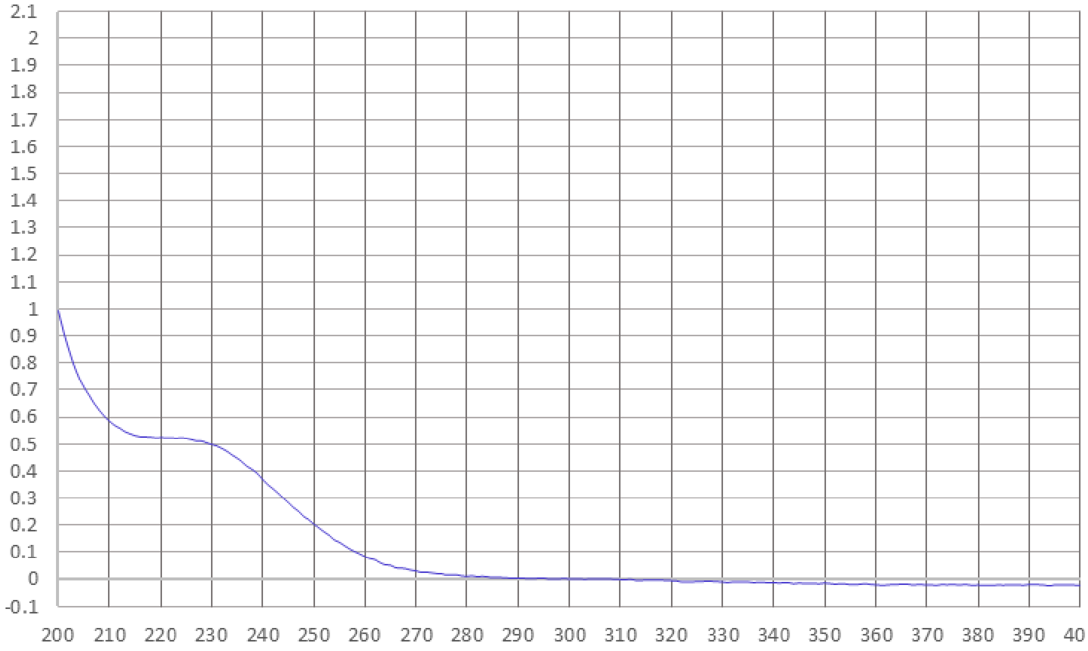

Figure S18.UV spectrum of compound 2.

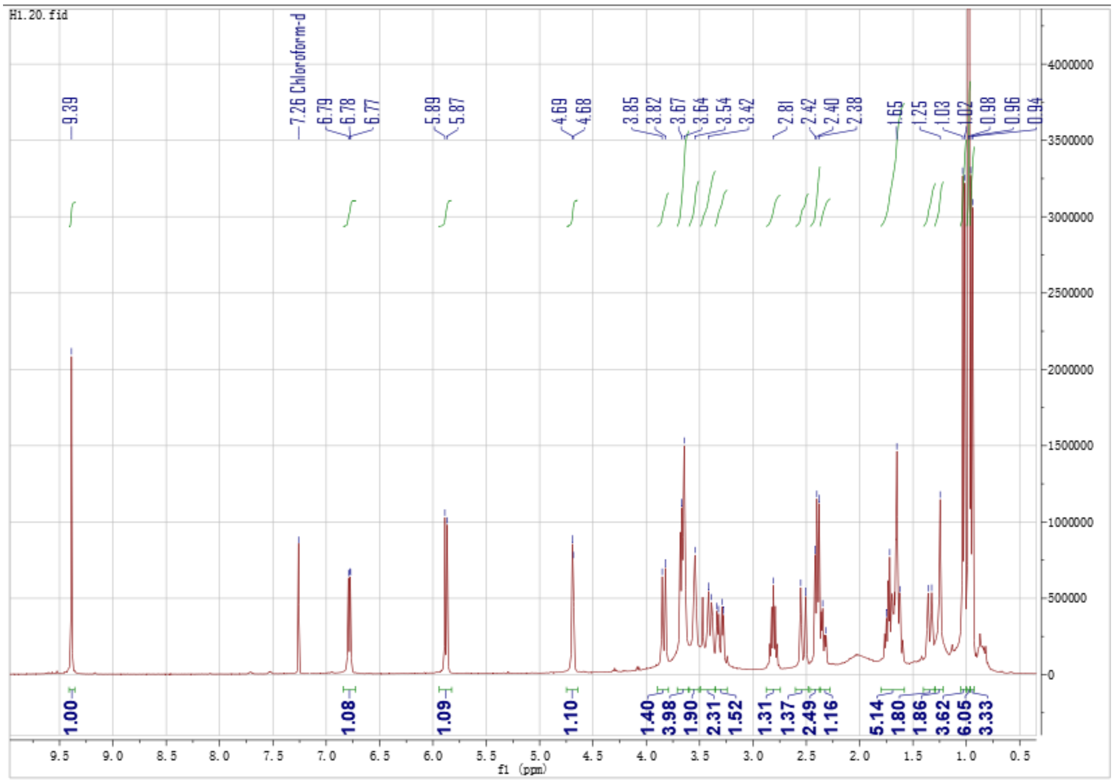

Figure S19. ^{1}**H NMR spectra of compound 3 in** $CDCl_3$

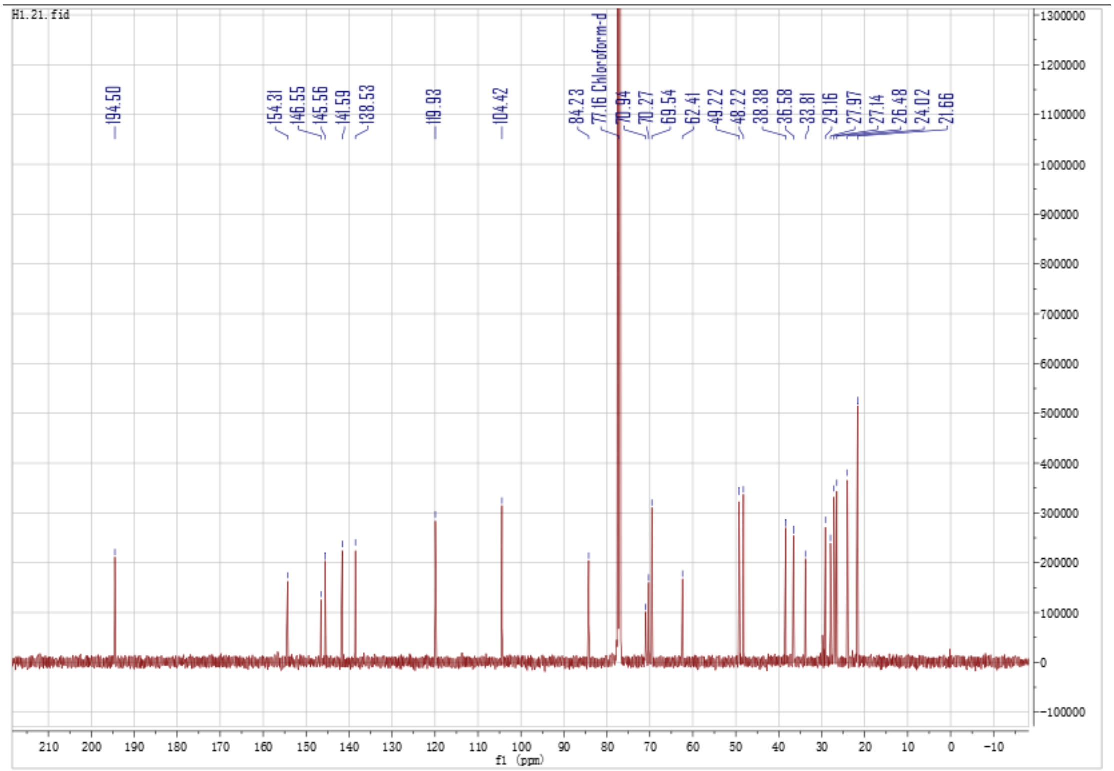

Figure S20. ^{13}**C spectra of compound 3 in** $CDCl_3$**.**

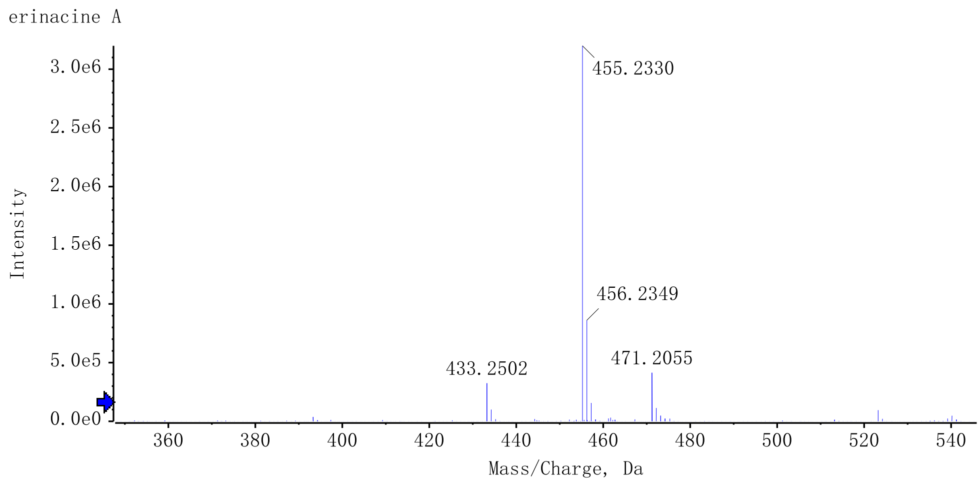

Figure S21.HRESI-MS spectrum of compound 3.

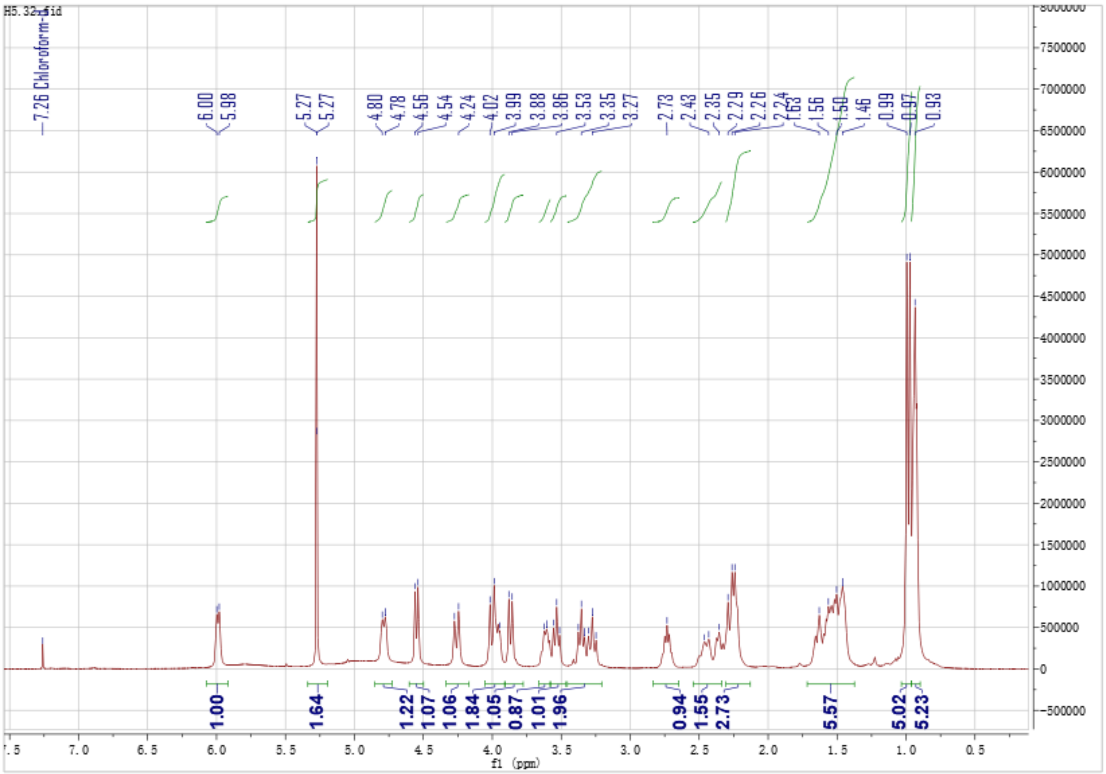

Figure S22. ^{1}H NMR spectra of compound 4 in $CDCl_3$

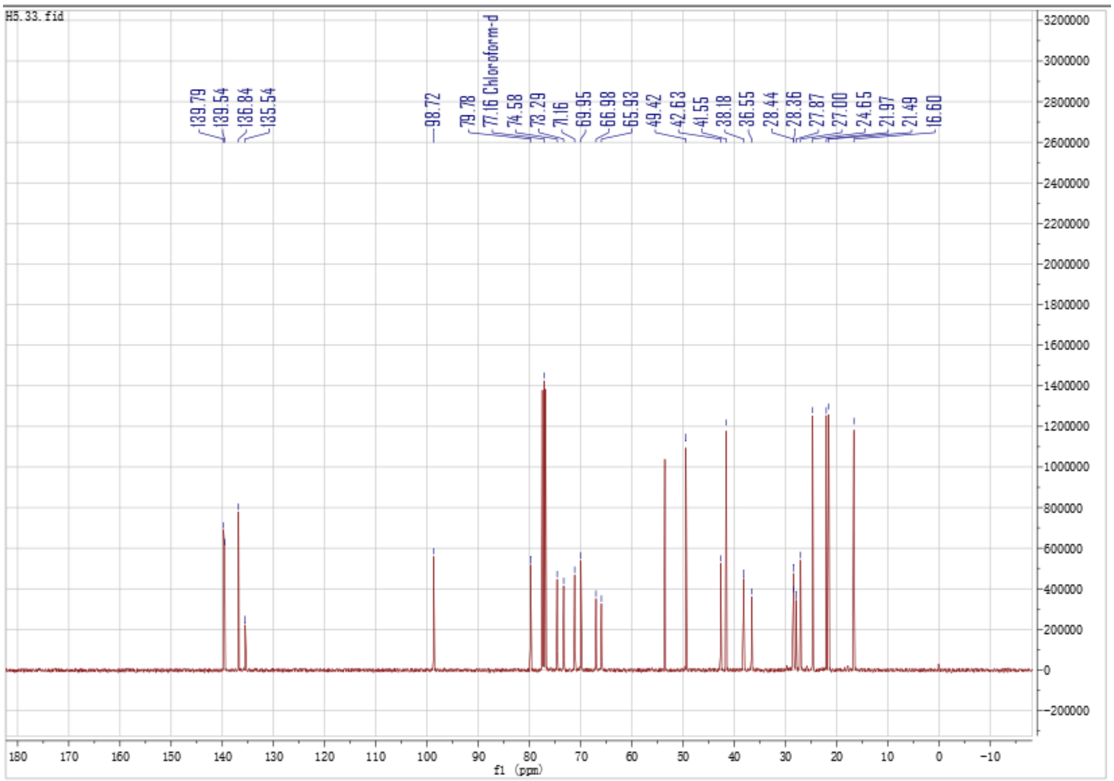

Figure S23. ^{13}C spectra of compound 4 in $CDCl_3$.

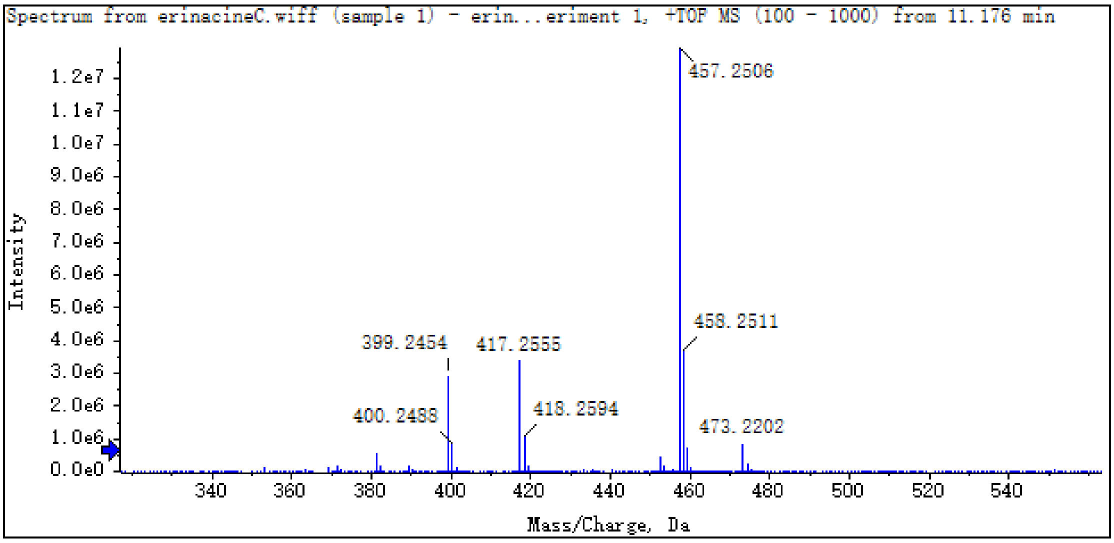

Figure S24.HRESI-MS spectrum of compound 4.

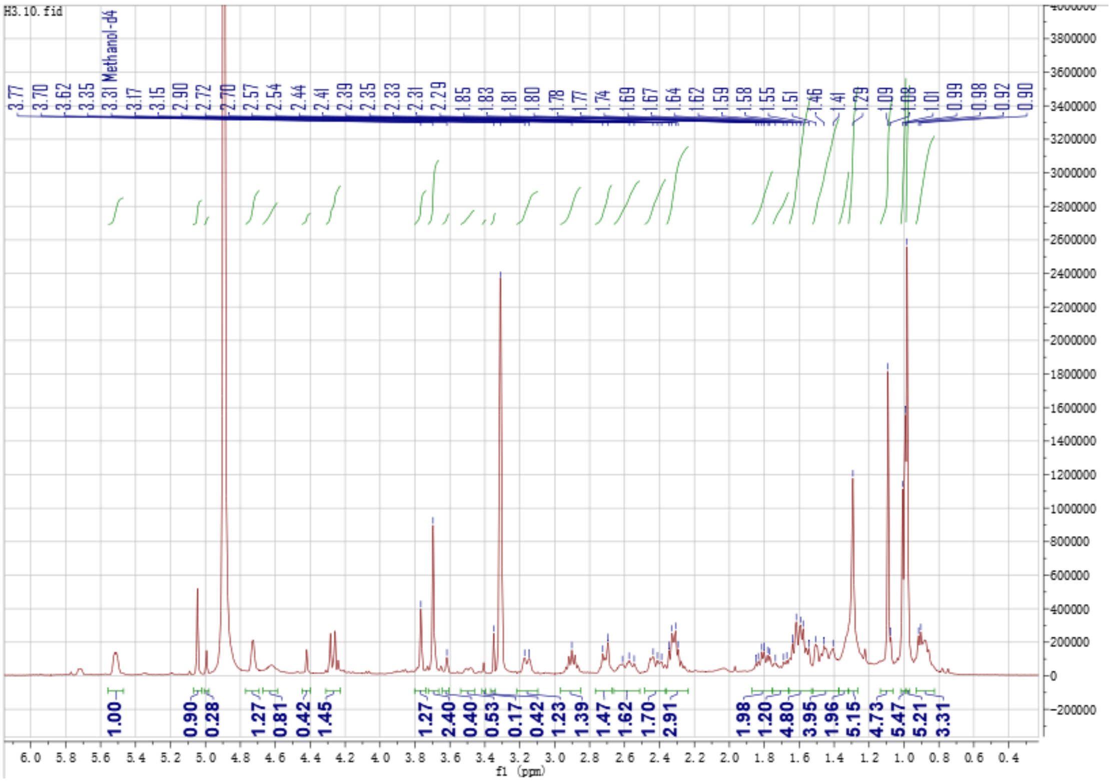

Figure S25. ^{1}H NMR spectra of compound 5 in CD3OD

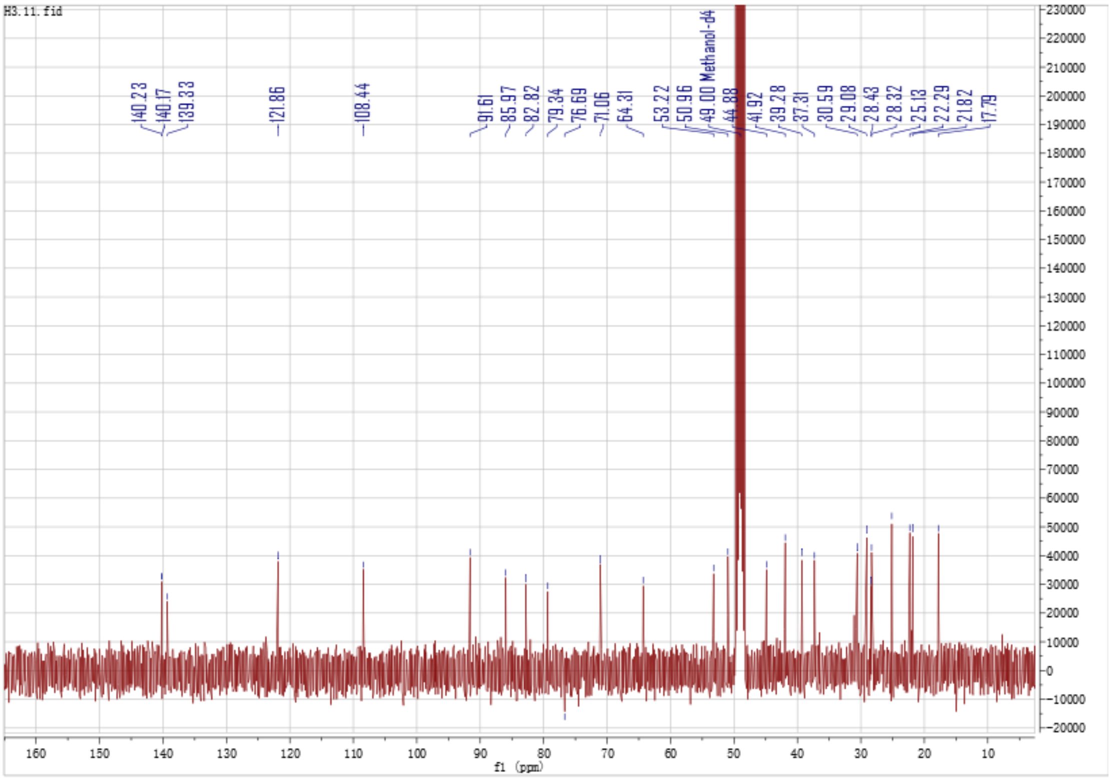

Figure S26. ^{13}C spectra of compound 5 in CD3OD.

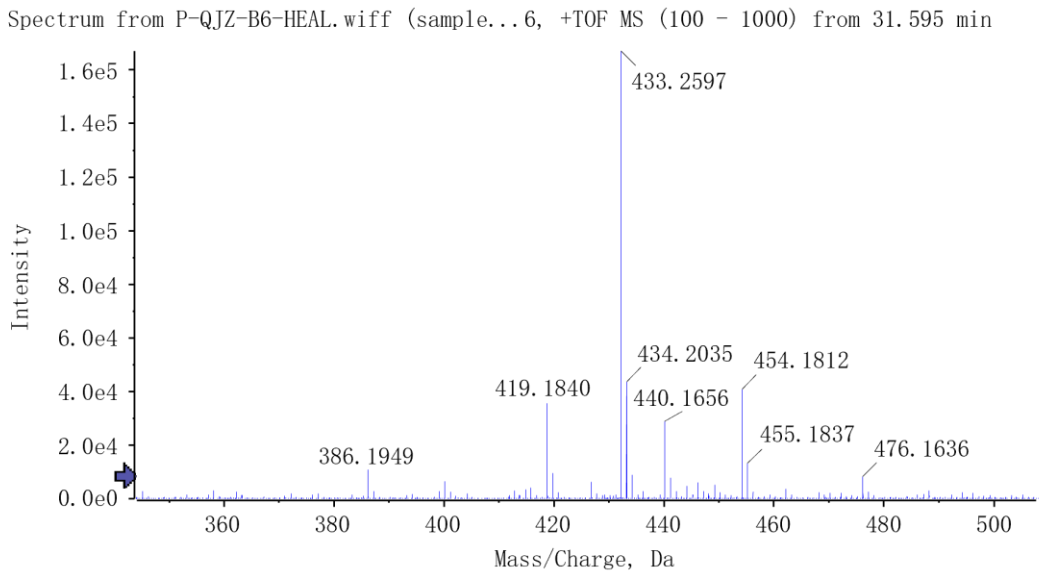

Figure S27.HRESI-MS spectrum of compound 5.

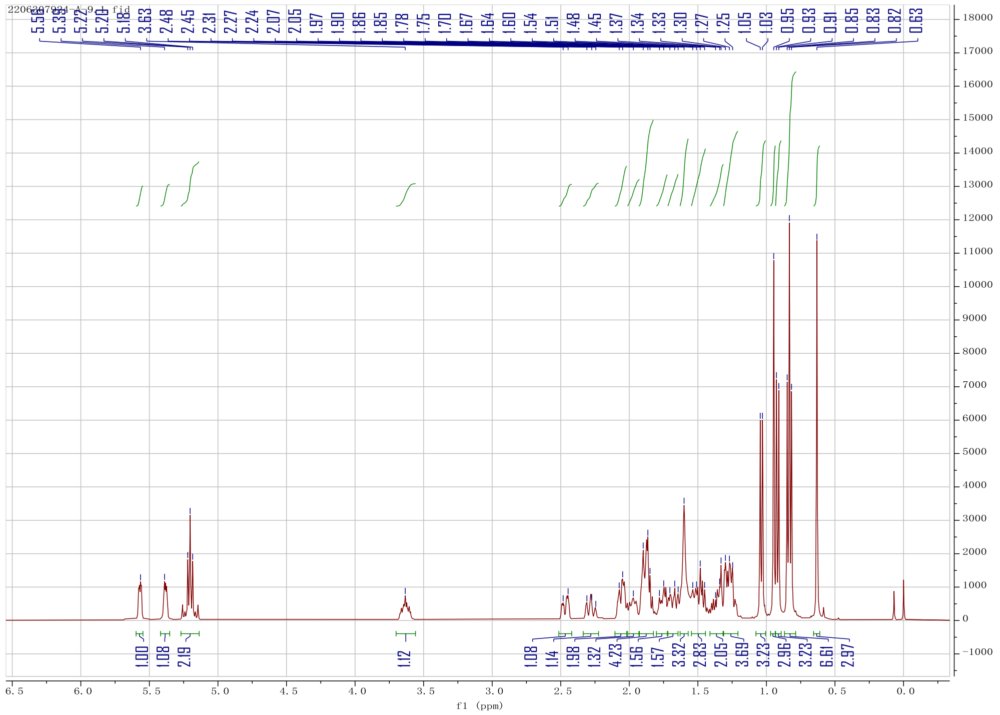

Figure S28. ^{1}**H NMR spectra of compound 6 in CDCl**$_3$**.**

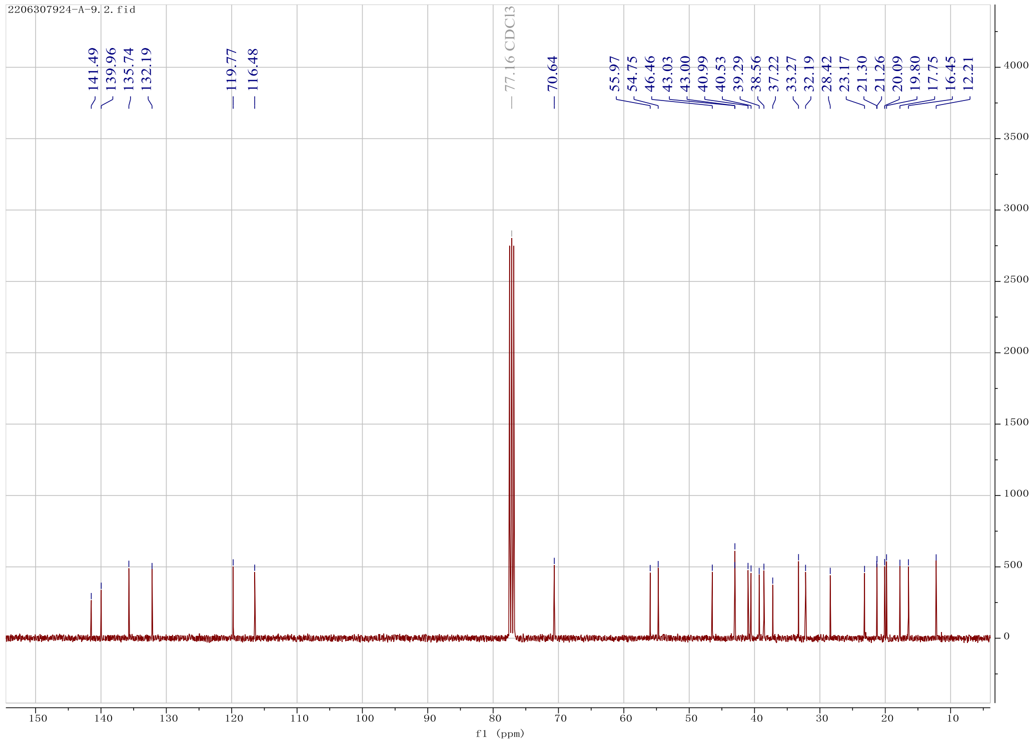

Figure S29. ^{13}**C spectra of compound 6 in CDCl**$_3$**.**

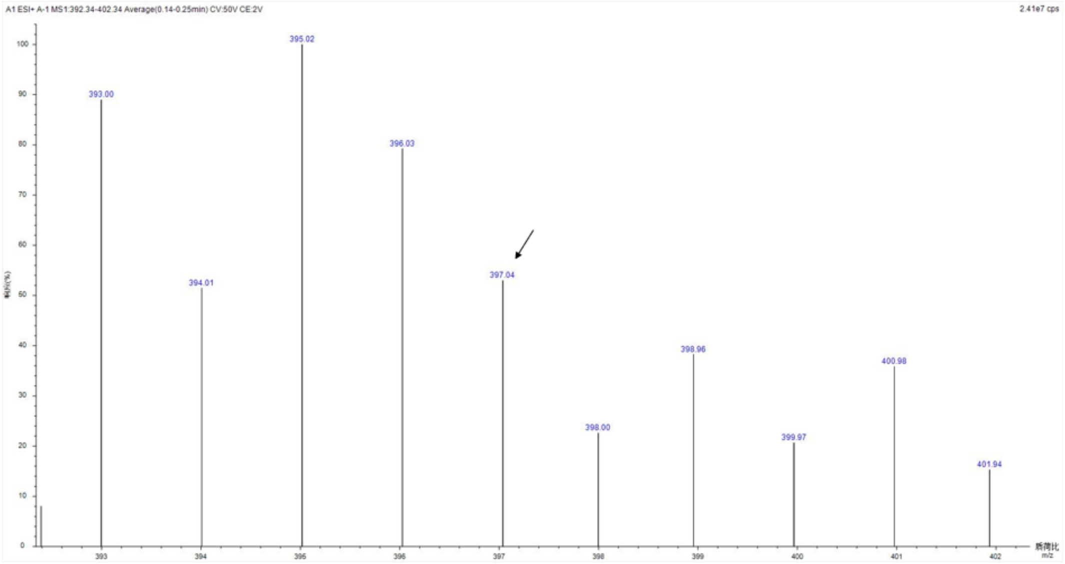

Figure S30. ESI-MS spectrum of compound 6.

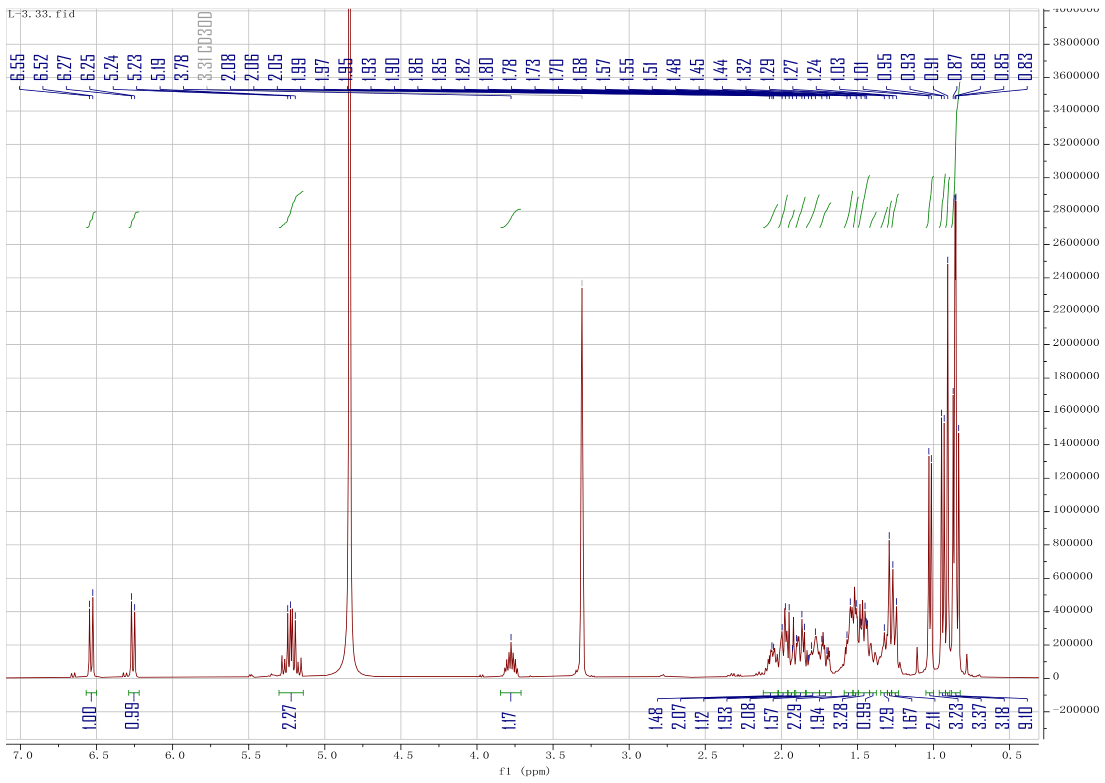

Figure S31. ^{1}H NMR spectra of compound 7 in CD_3OD.

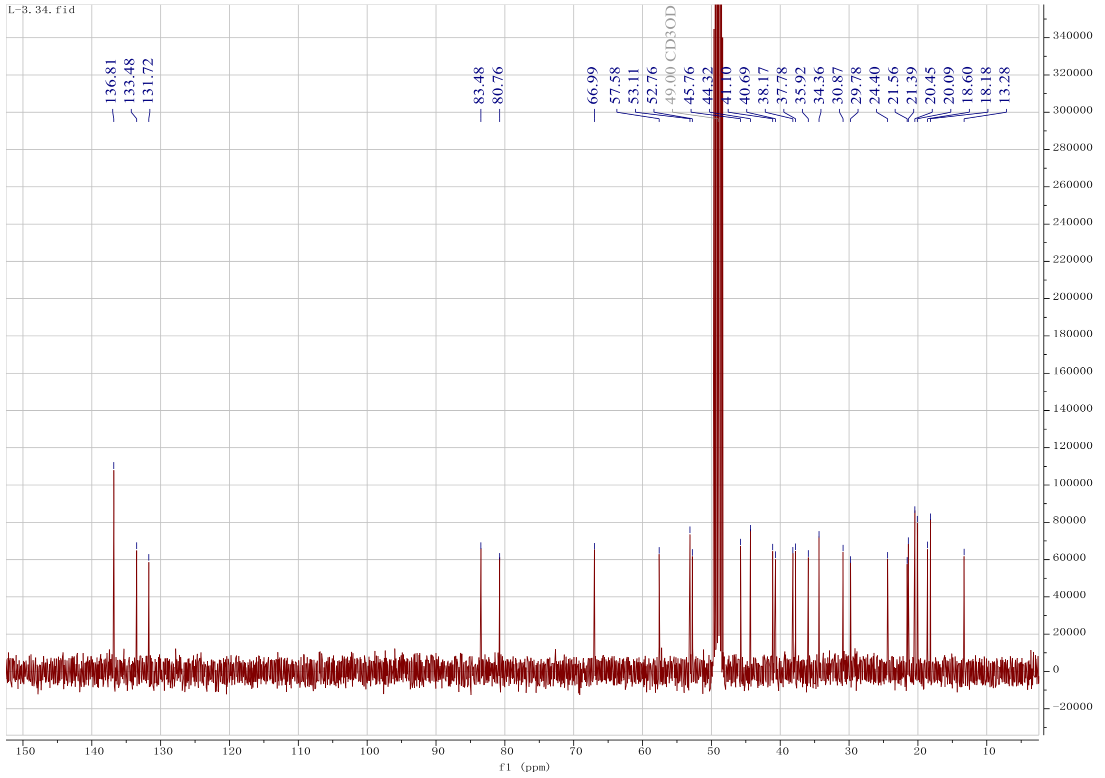

Figure S32. ^{13}C spectra of compound 7 in CD_3OD.

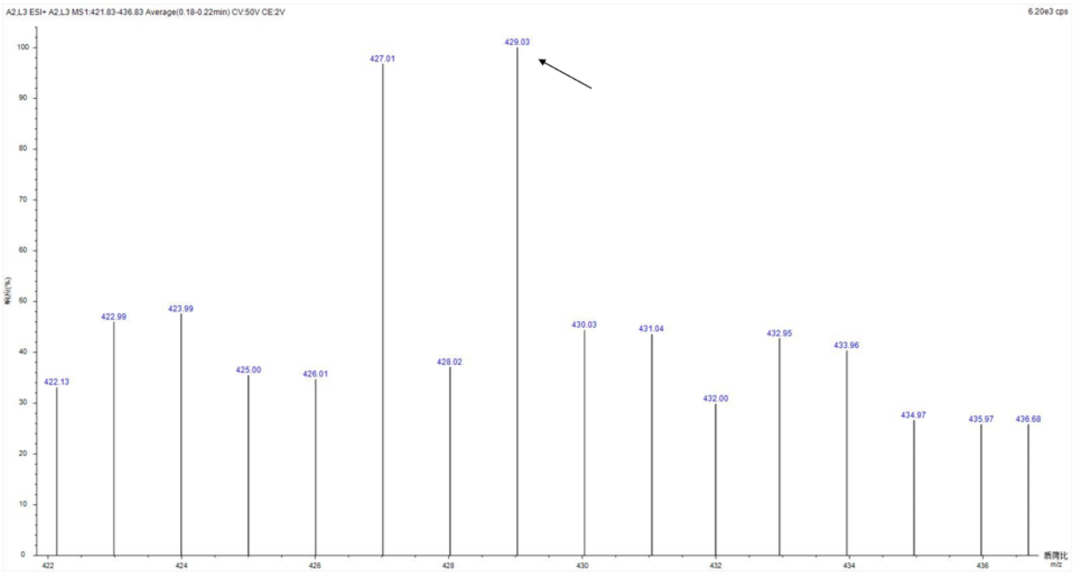

Figure S33. ESI-MS spectrum of compound 7.

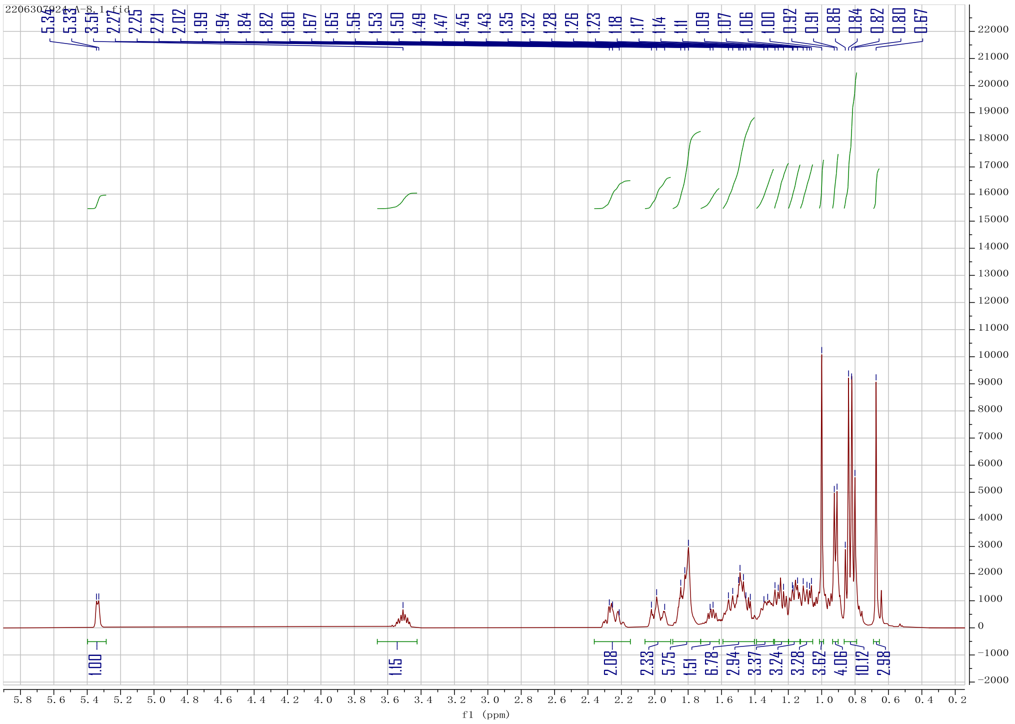

Figure S34. ^{1}H NMR spectra of compound 8 in $CDCl_3$.

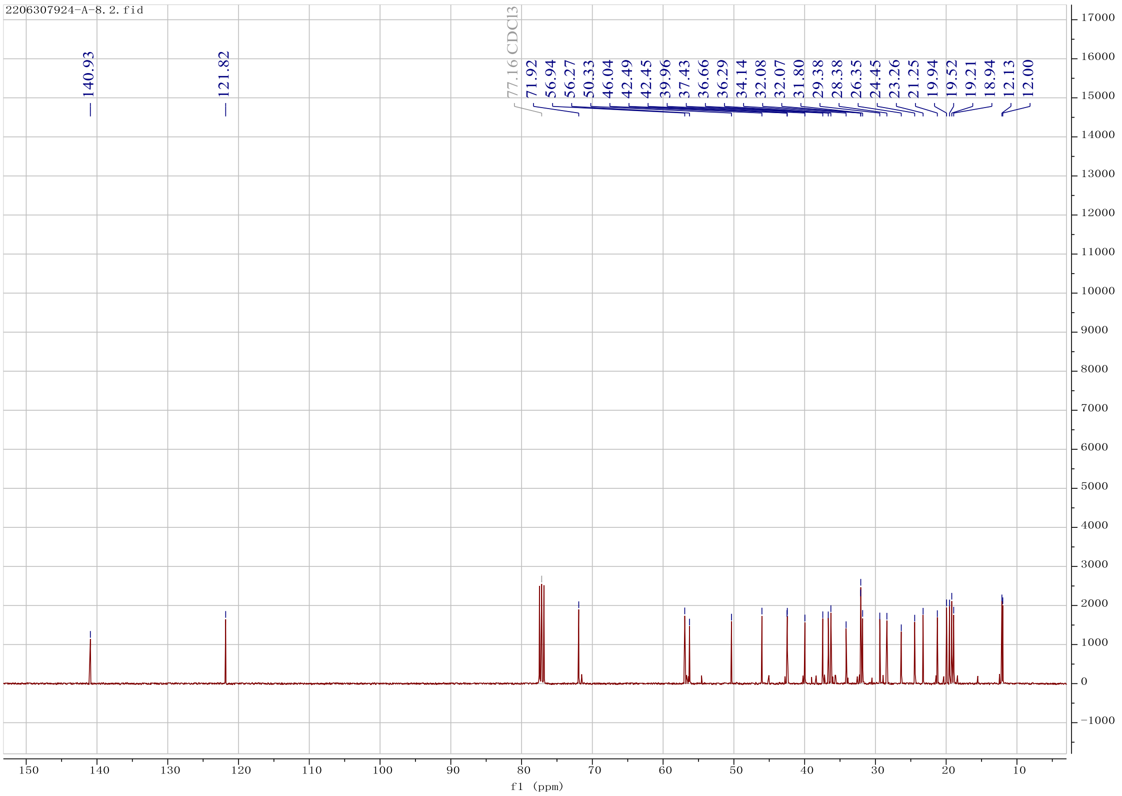

Figure S35. ^{13}C spectra of compound 8 in $CDCl_3$.

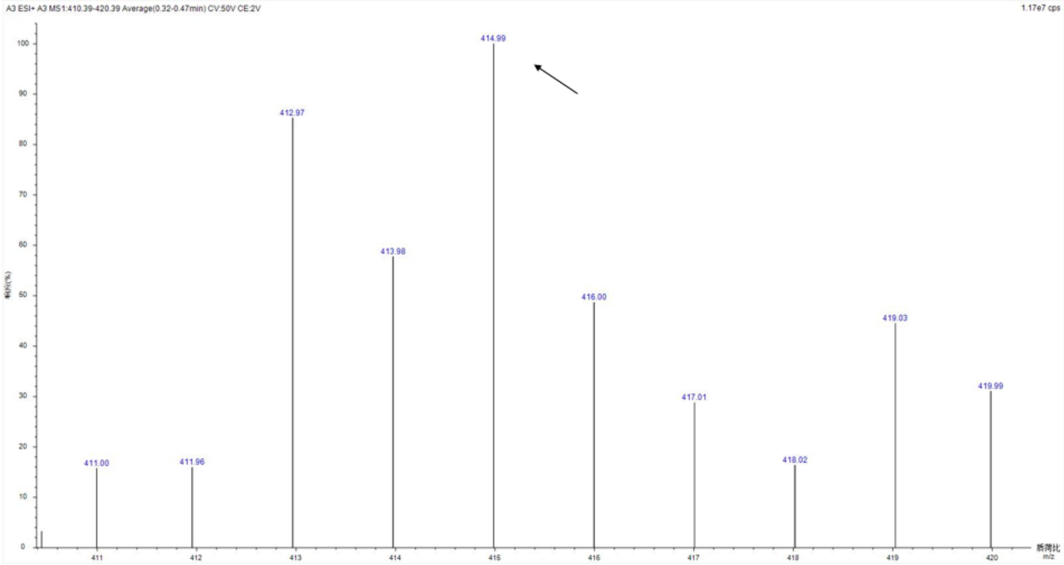

Figure S36. ESI-MS spectrum of compound 8.

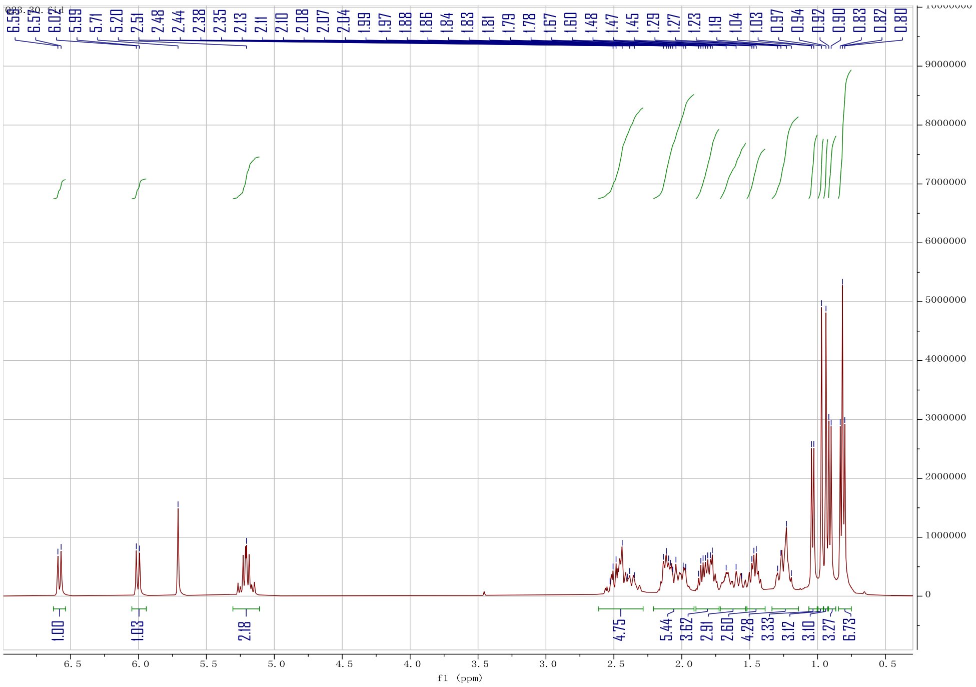

Figure S37. ^{1}H NMR spectra of compound 9 in $CDCl_3$.

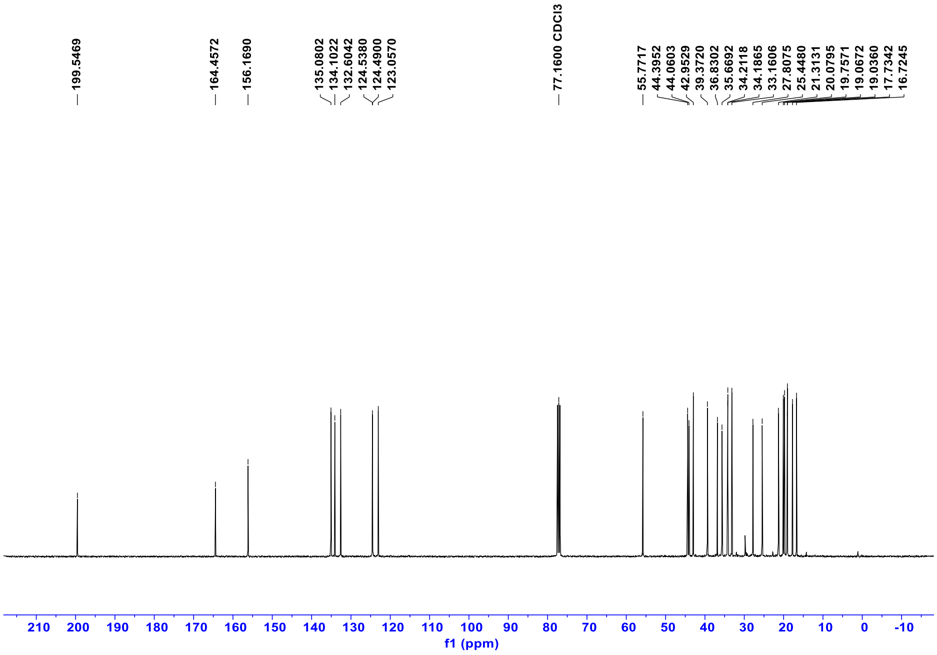

Figure S38. ^{13}C spectra of compound 9 in $CDCl_3$.

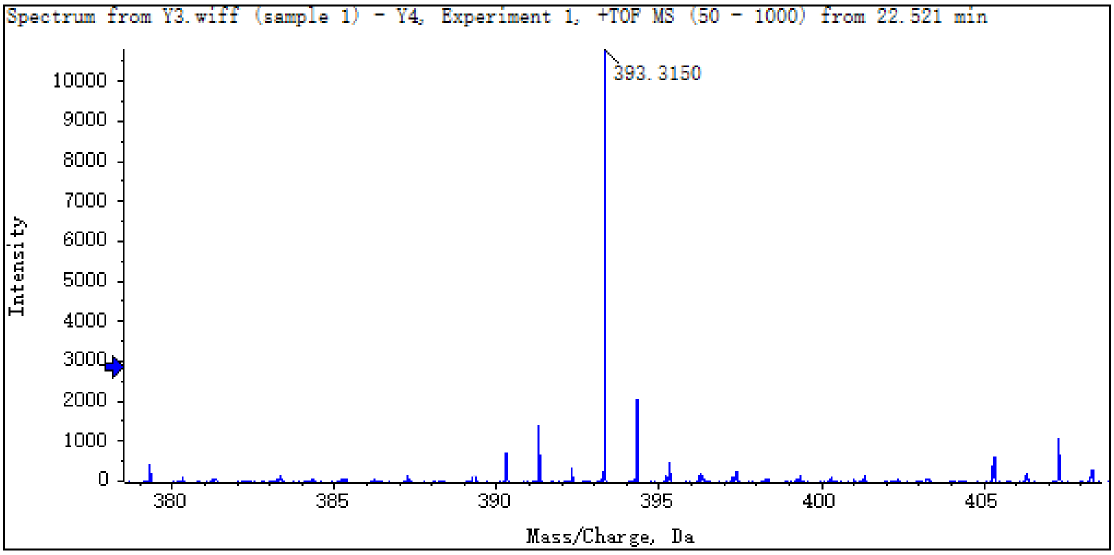

Figure S39.HRESI-MS spectrum of compound 9.

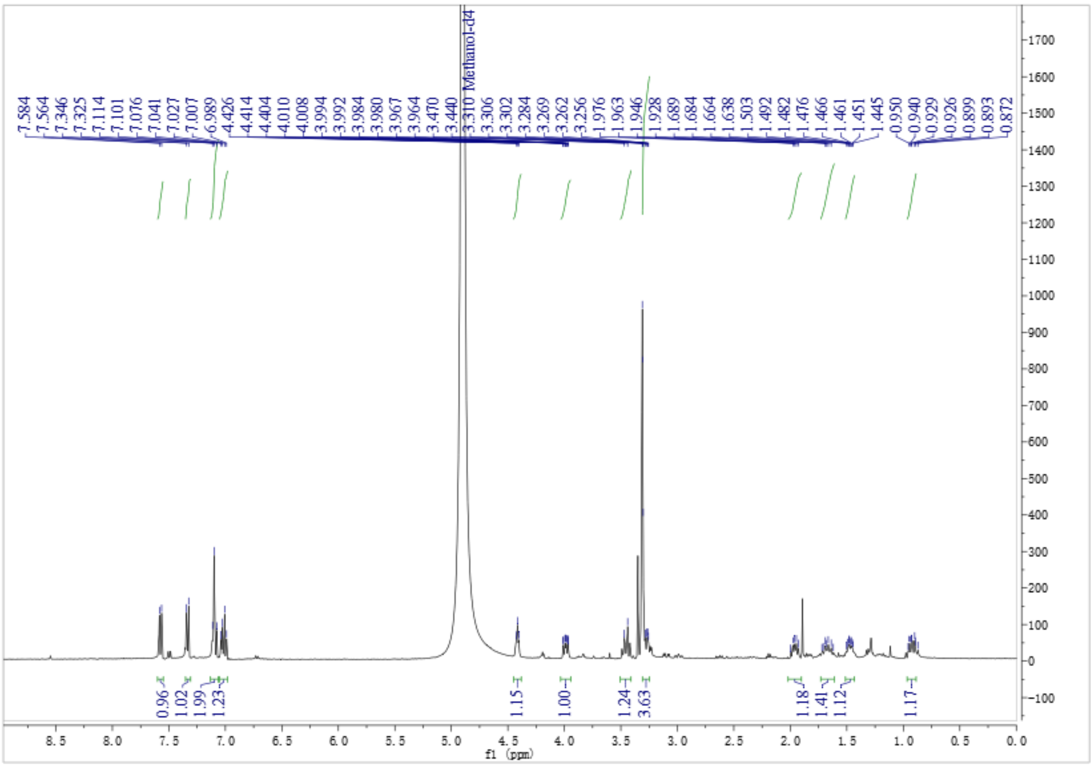

Figure S40. ¹H NMR spectra of compound 10 in CD$_3$OD.

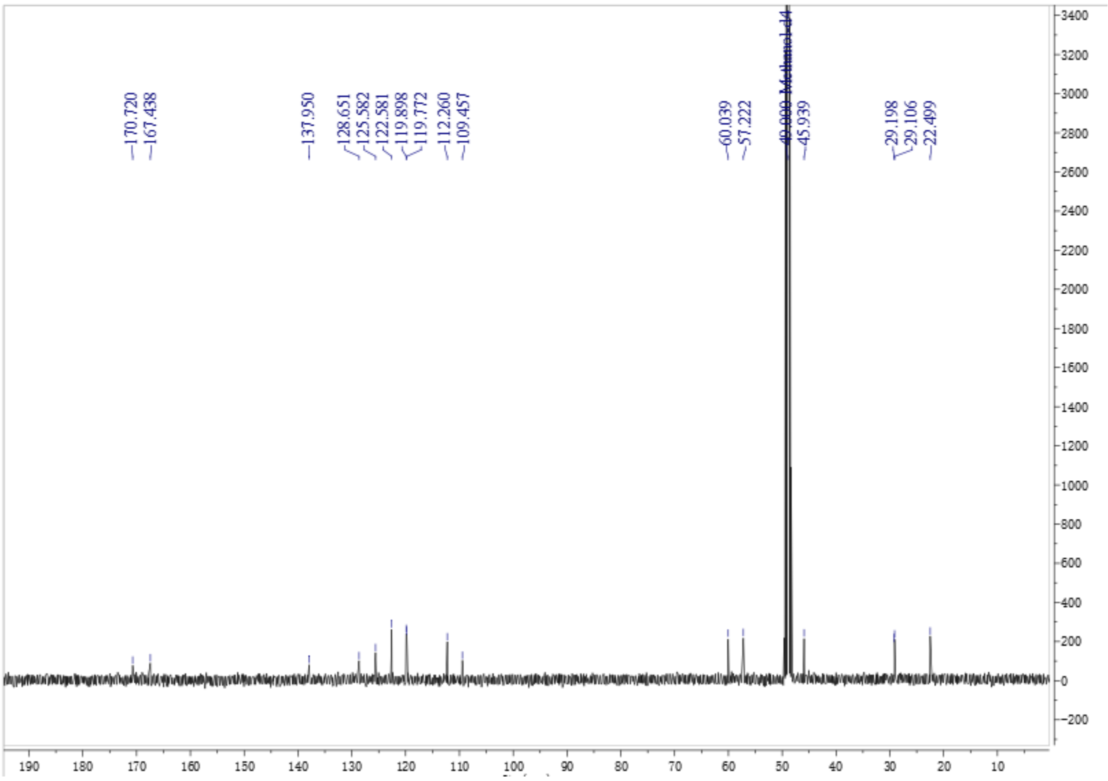

Figure S41. ¹³C spectra of compound 10 in CD$_3$OD.

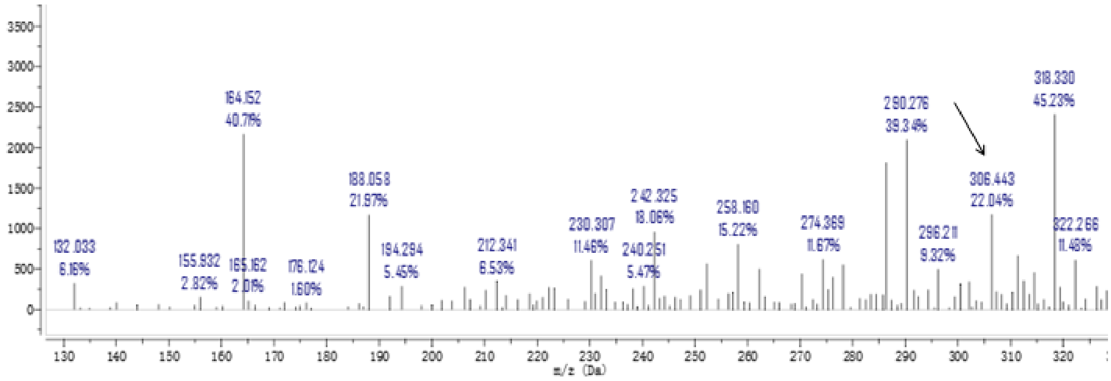

Figure S42.ESI-MS spectrum of compound 10.

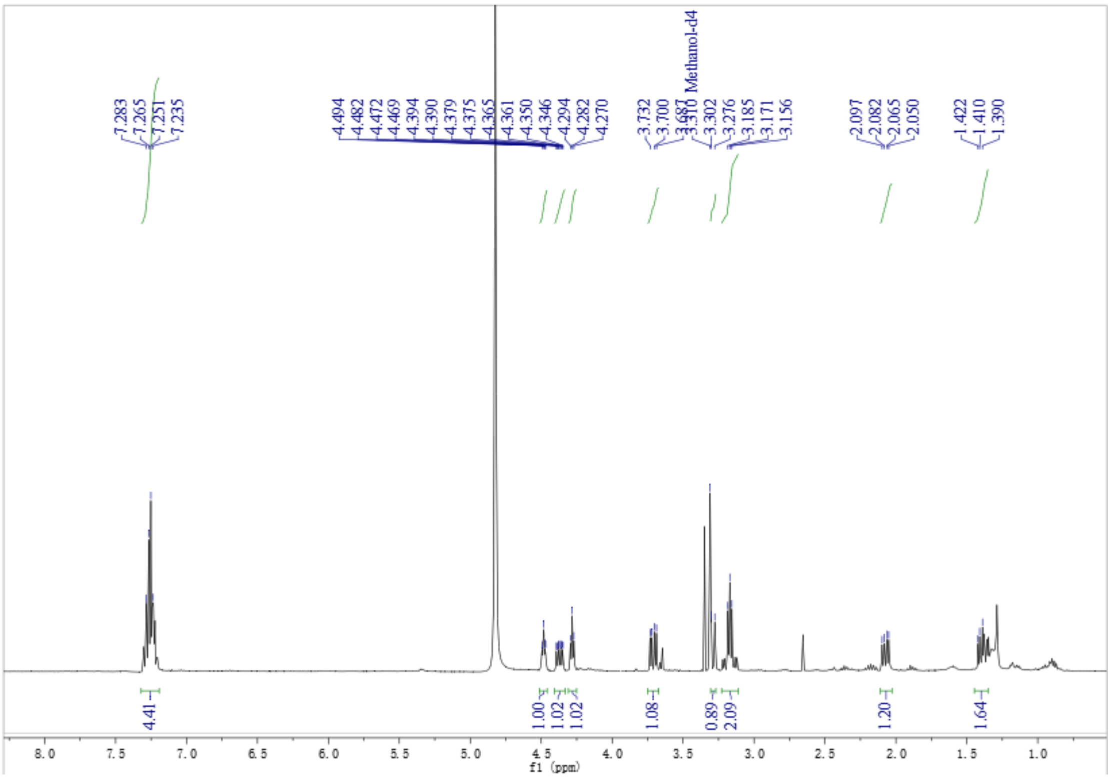

Figure S43. **^{1}H NMR spectra of compound 11 in CD_3OD.**

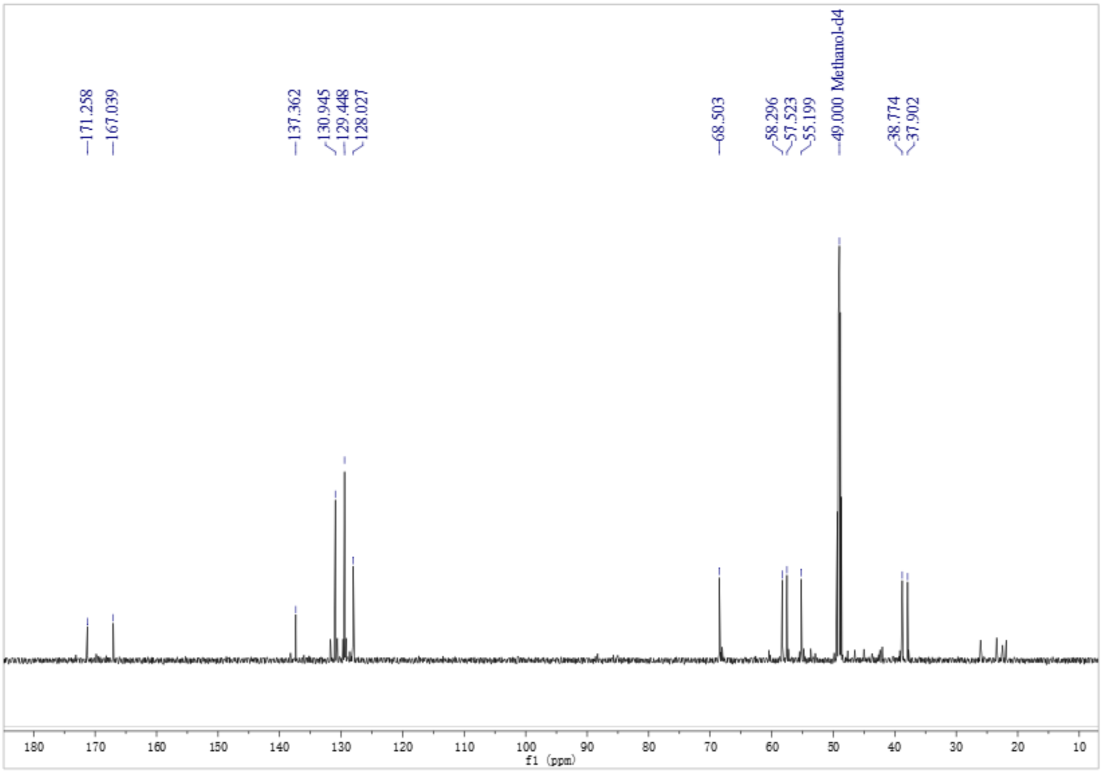

Figure S44. **^{13}C spectra of compound 11 in CD_3OD.**

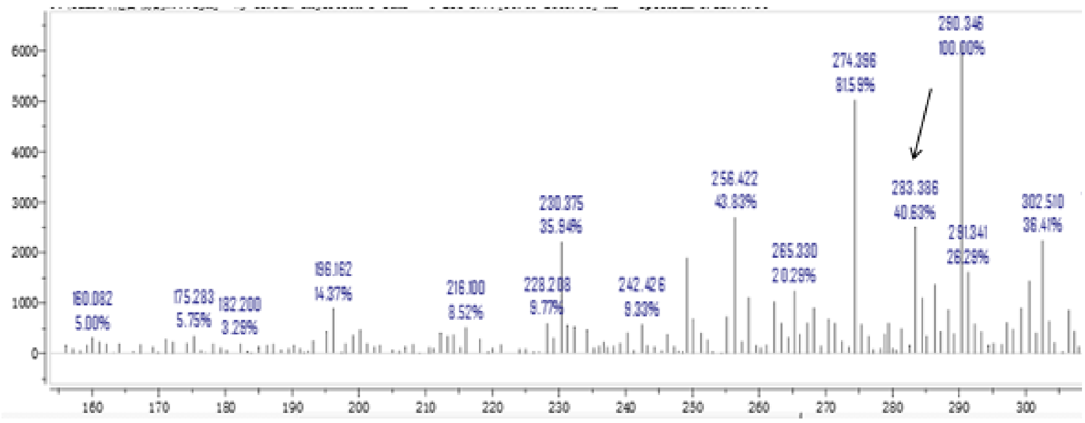

Figure S45.ESI-MS spectrum of compound 11.

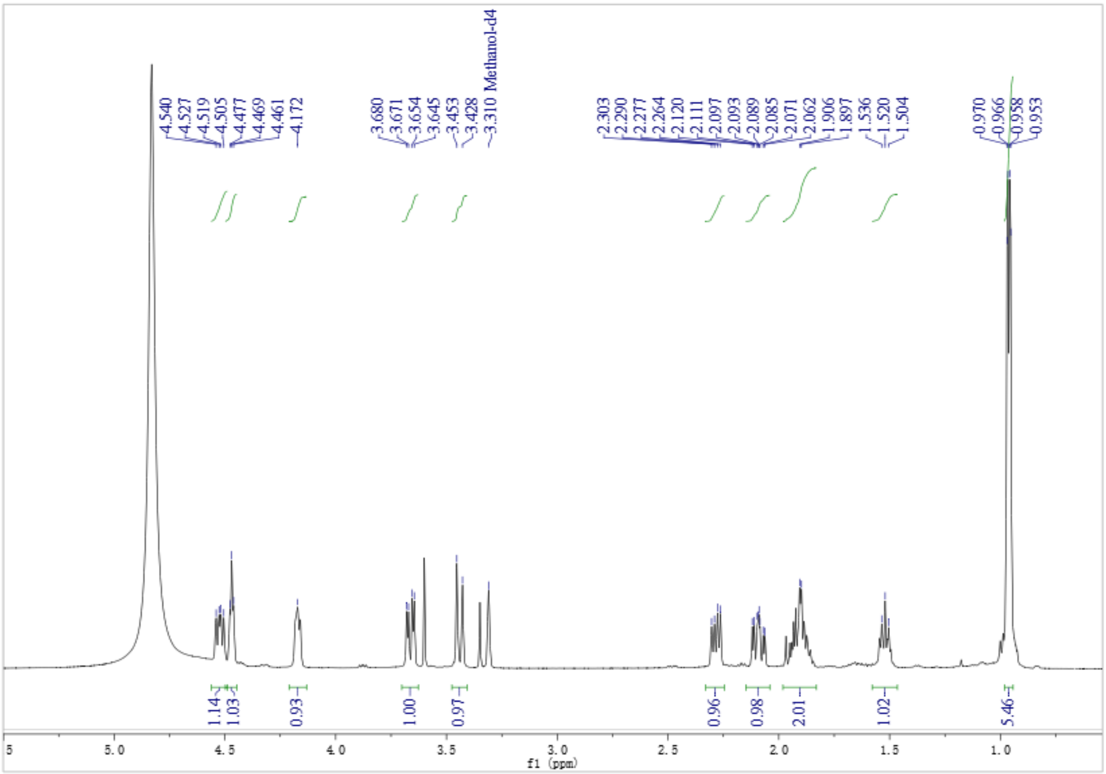

Figure S46. ^{1}H NMR spectra of compound 12 in CD_3OD.

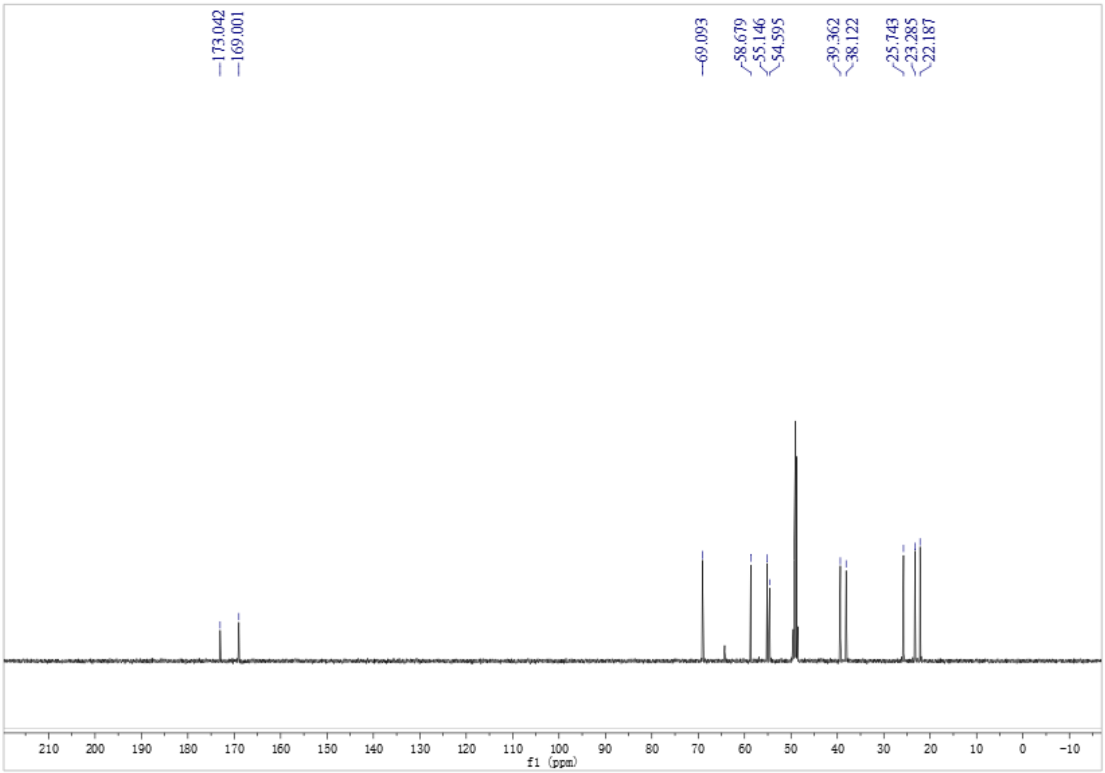

Figure S47. ^{13}C spectra of compound 12 in CD_3OD.

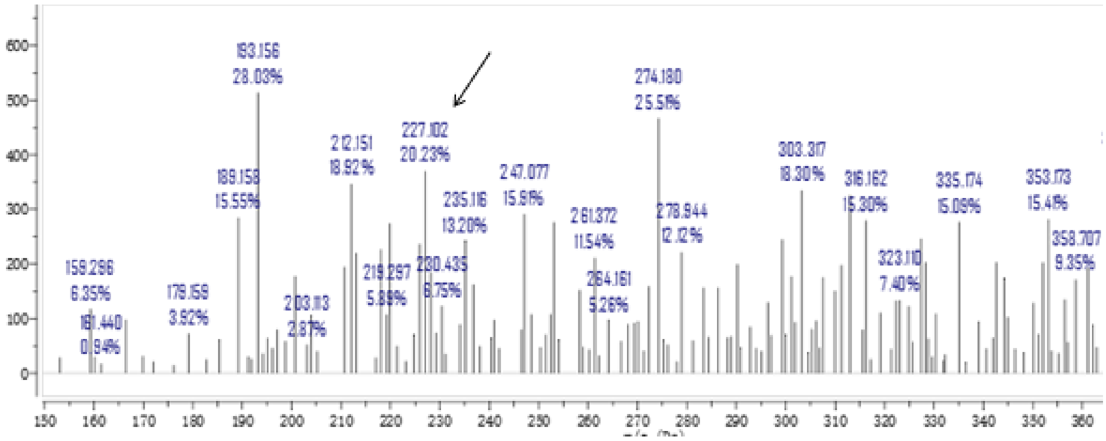

Figure S48.ESI-MS spectrum of compound 12.

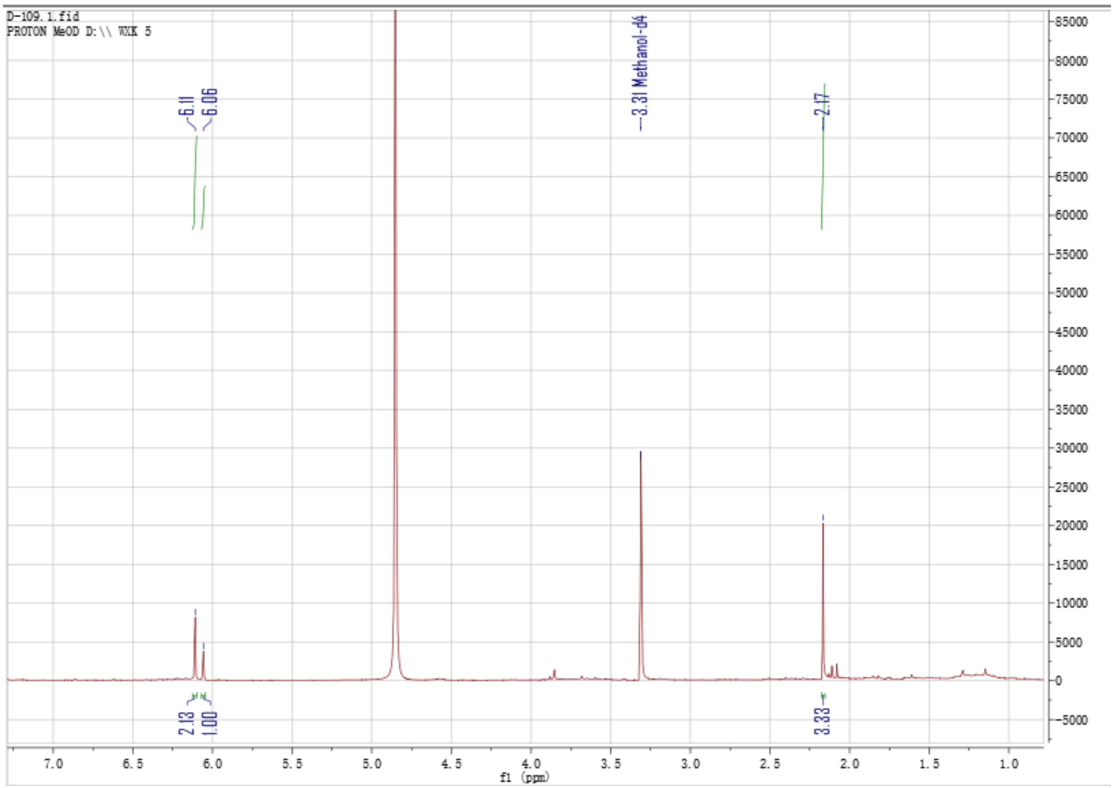

Figure S49. ^{1}H NMR spectra of compound 13 in CD_3OD.

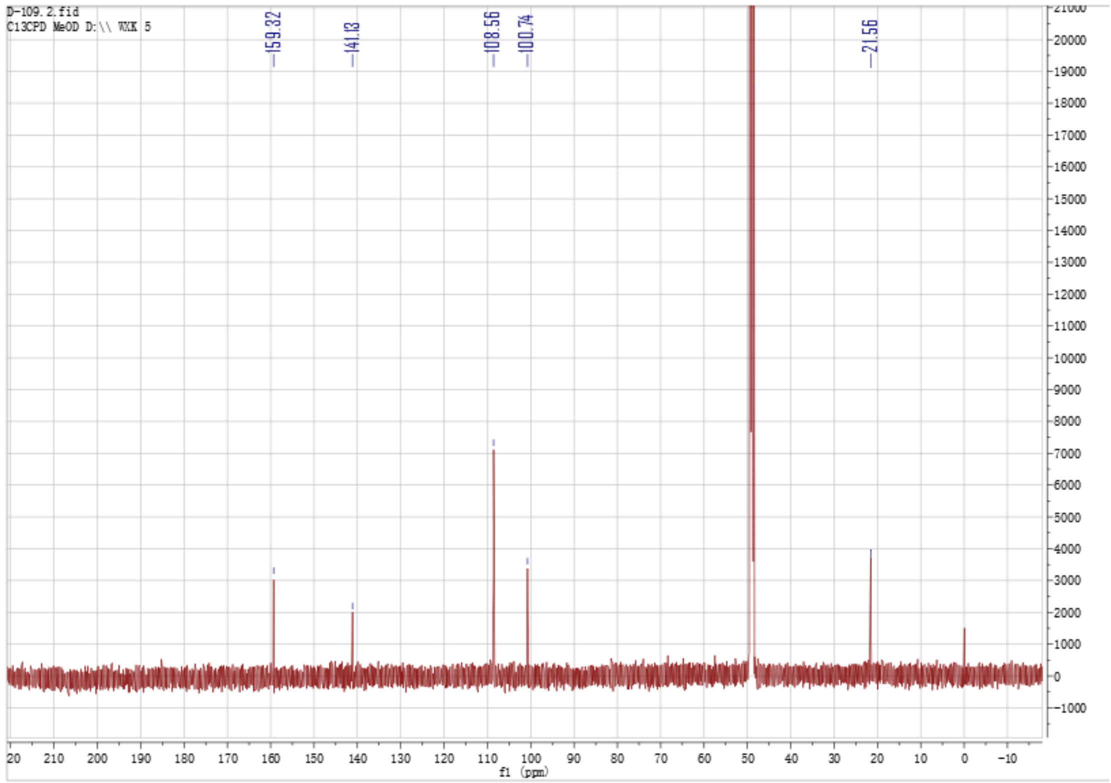

Figure S50. ^{13}C spectra of compound 13 in CD_3OD.

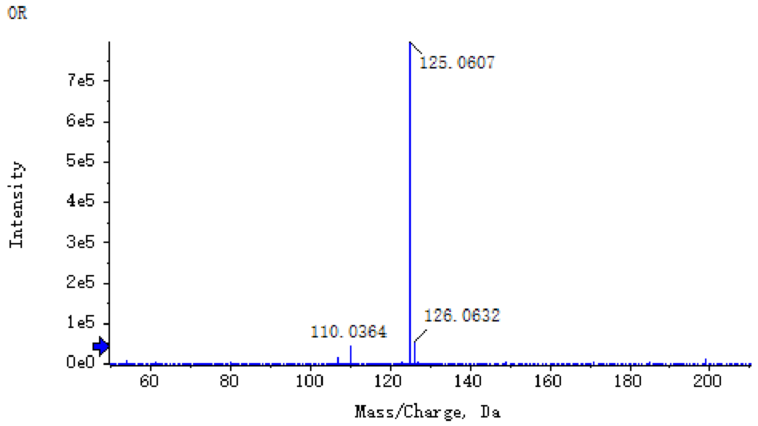

Figure S51.HRESI-MS spectrum of compound 13.

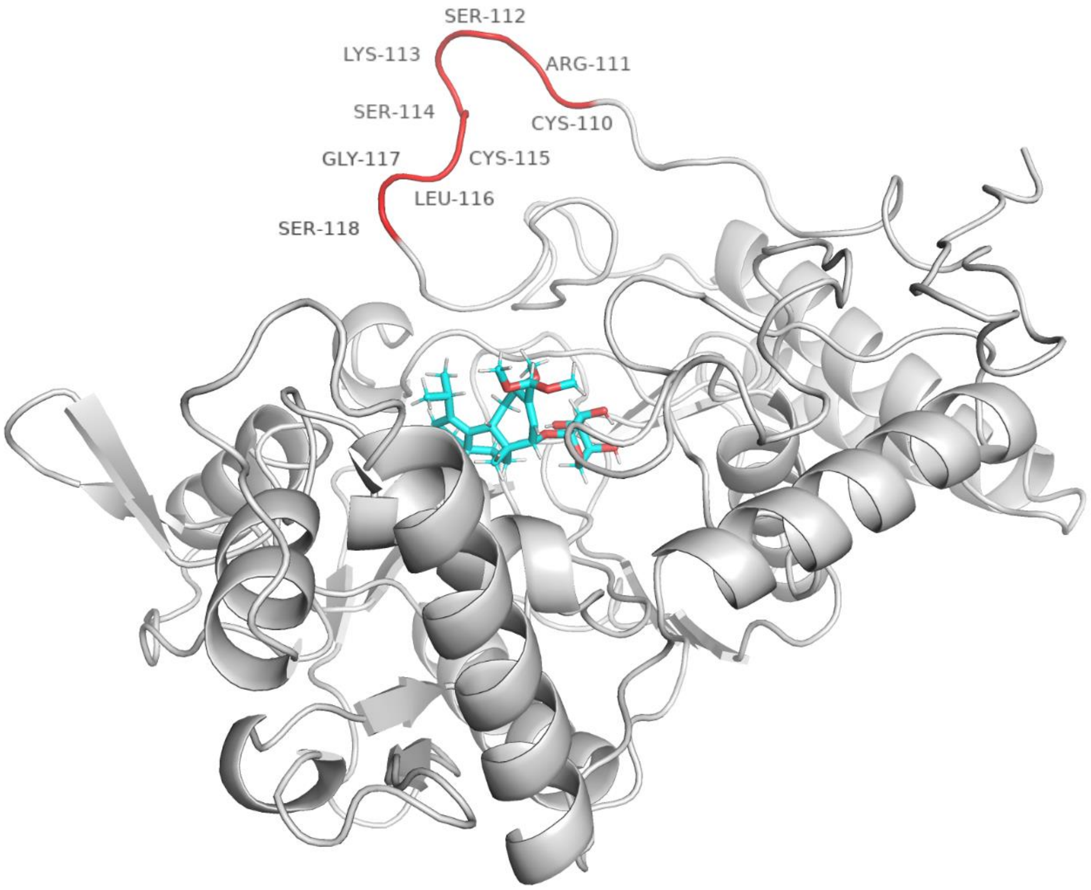

Figure S52.Region of protein instability identified by RMSF analysis.

Table S1. IC_{50} data of 1-9 inhibited the LPS-induced NO production in culture medium.

Compounds	IC_{50} (μM)
1	10.14±0.14
2	5.82±0.18
3	31.44±1.39
4	29.22±0.57
5	23.89±0.83
6	>40
7	>40
8	>40
9	>40
Quercetin	15.88±0.12
