# Supplementary material for: Unprecedented Neoverrucosane and Cyathane Diterpenoids with Anti-Neuroinflammatory Activity from Cultures of the Culinary-Medicinal Mushroom Hericium erinaceus
Source: Molecules. 2023 Aug 31;28(17):6380. doi: 10.3390/molecules28176380 (PMC10489798; doi:10.3390/molecules28176380)
Supplement: Supplementary file 1 [file molecules-28-06380-s001.zip › The original PAGE graph of WB.pdf]

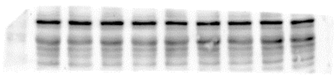

NF-KB P65， 1st band: blank, 2nd band: LPS-induced, the five band: compound **2** treated.

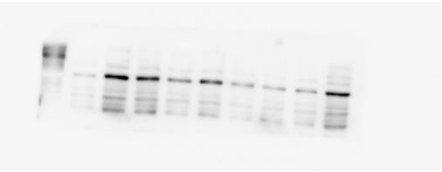

P-NF-KB P65, 1st band: blank, 2nd band: LPS-induced, the five band: compound **2** treated.

iNOS, 1st band: blank, 2nd band: LPS-induced, the five band: compound **2** treated.

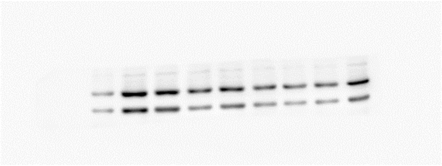

COX2, 1st band: blank, 2nd band: LPS-induced, the five band: compound **2** treated.

TLR4, 1st band: blank, 2nd band: LPS-induced, the five band: compound **2** treated.

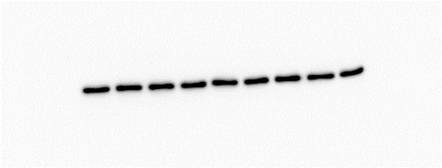

GAPDH, 1st band: blank, 2nd band: LPS-induced, the five band: compound **2** treated.
